# Chemical Modifications Suppress Anharmonic Effects in the Lattice Dynamics of Organic Semiconductors

**DOI:** 10.1021/acsmaterialsau.2c00020

**Published:** 2022-07-05

**Authors:** Maor Asher, Rémy Jouclas, Marco Bardini, Yael Diskin-Posner, Nitzan Kahn, Roman Korobko, Alan R. Kennedy, Lygia Silva de Moraes, Guillaume Schweicher, Jie Liu, David Beljonne, Yves Geerts, Omer Yaffe

**Affiliations:** † Department of Chemical and Biological Physics, Weizmann Institute of Science, Rehovot 76100, Israel; ‡ Laboratoire de Chimie des Polymères, Université Libre de Bruxelles (ULB), 1050 Brussels, Belgium; § Laboratory for Chemistry of Novel Materials, University of Mons, 7000 Mons, Belgium; ∥ Chemical Research Support, Weizmann Institute of Science, Rehovot 76100, Israel; ⊥ Department of Pure and Applied Chemistry, University of Strathclyde, Glasgow G1 1XL, United Kingdom; # International Solvay Institutes for Physics and Chemistry, 1050 Brussels, Belgium

**Keywords:** small-molecule organics semiconductors, organic crystals, lattice dynamics, temperature and polarization-dependent Raman spectroscopy, vibrational anharmonicity, density functional theory, temperature-dependent X-ray diffraction

## Abstract

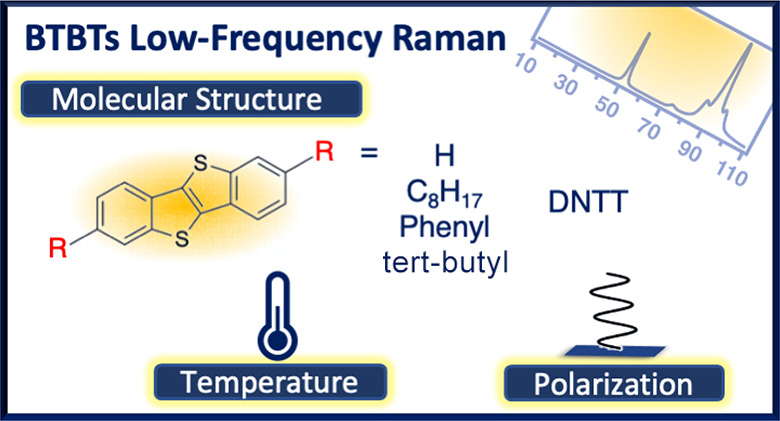

The lattice dynamics of organic semiconductors has a significant role in determining their electronic and mechanical properties. A common technique to control these macroscopic properties is to chemically modify the molecular structure. These modifications are known to change the molecular packing, but their effect on the lattice dynamics is relatively unexplored. Therefore, we investigate how chemical modifications to a core [1]benzothieno[3,2-*b*]benzothiophene (BTBT) semiconducting crystal affect the evolution of the crystal structural dynamics with temperature. Our study combines temperature-dependent polarization-orientation (PO) low-frequency Raman measurements with first-principles calculations and single-crystal X-ray diffraction measurements. We show that chemical modifications can indeed suppress specific expressions of vibrational anharmonicity in the lattice dynamics. Specifically, we detect in BTBT a gradual change in the PO Raman response with temperature, indicating a unique anharmonic expression. This anharmonic expression is suppressed in all examined chemically modified crystals (ditBu-BTBT and diC8-BTBT, diPh-BTBT, and DNTT). In addition, we observe solid–solid phase transitions in the alkyl-modified BTBTs. Our findings indicate that π-conjugated chemical modifications are the most effective in suppressing these anharmonic effects.

## Introduction

Contemporary theoretical calculations of macroscopic properties of organic solids at finite temperatures often use the harmonic approximation to describe their lattice dynamics.^1−9^ This means that the thermal fluctuations of the molecules are assumed to be small and, therefore, follow a parabolic potential surface. In other words, the vibrational dynamics is described as a linear system of noninteracting normal modes (i.e., phonons). However, in general, for any material, this approach fails to explain by definition important physical phenomena, such as thermal expansion, the temperature dependence of phonon frequencies, phonon lifetimes, phase transitions, and thermal conductivity.^10^

The phenomena mentioned above are macroscopic manifestations of the interactions between phonons. This means that the potential surface of the molecular displacements is not parabolic but includes higher-order terms (e.g., cubic and quartic).^11^ These are known as the anharmonic terms. Accordingly, each phenomenon that the harmonic approximation fails to describe is considered a manifestation of vibrational anharmonicity.^10,11^

Organic crystals which are held together primarily by weak van der Waals forces usually exhibit strongly anharmonic thermal fluctuations.^9,12,13^ Indeed, they often exhibit large thermal expansion^14^ and isothermal compressibility^15^ constants, strong temperature dependence of the frequency and lifetime of their lattice vibrations,^16−18^ and solid–solid phase transitions.^19−21^ Moreover, the lattice dynamics of organic semiconductors plays a key role in determining their electronic,^8,9,22−24^ optical,^25−27^ thermal,^28,29^ and mechanical^30,31^ properties. For instance, anharmonic lattice dynamics can affect their electron–phonon coupling^8,22^ and the polaronic character.^25^ Therefore, a comprehensive understanding of anharmonic effects at finite temperatures is essential for the effective implementation of organic crystals in applications.

Currently, the most common approach to include anharmonic effects is the quasi-harmonic approximation (QHA).^30−34^ In the QHA, the temperature dependence of the vibrational frequencies is calculated for each given temperature to account only for thermal expansion.^10^ Notably, these frequencies are later used within the harmonic model for further calculations, meaning any effect that involves phonon lifetimes is not captured. Other theoretical approximated frameworks, which include a correction to the interatomic potential due to its anharmonic nature, are either not accurate for organic materials^35^ or computationally expensive.^36^

In the field of organic electronics, modification of the molecular structure is a common strategy for crystal engineering aimed to optimize the electronic properties.^37−42^ Recent studies have shown that such chemical modifications strongly affect the lattice dynamics.^9,43,44^ These studies have suggested that introducing side chains to a molecule can suppress large-amplitude motion, thereby reducing the dynamic disorder and improving charge mobility. Therefore, for the rational design of organic semiconductors, it is vital to understand how different types of chemical modifications affect the lattice dynamics and to define the preferred behavior at room temperature.

In this study, we investigate the relationship between the molecular structure and the evolution with temperature of the structural dynamics. Our approach combines temperature-dependent, polarization-orientation (PO) Raman scattering at the THz (i.e., low-frequency) range with first-principles simulations and single-crystal X-ray diffraction (SC-XRD) to study the structural dynamics of [1]benzothieno[3,2-*b*]benzothiophene (**BTBT**) semiconducting crystal and its derivatives (Table 1). From the comparison, we learn that different chemical modifications can suppress specific expressions of vibrational anharmonicity but can also change the type of anharmonic expressions in the crystals. In the following, we first describe the evolution of the structural dynamics with temperature of BTBT as it is the parent molecule. We then describe the structural dynamics of its derivatives in comparison to BTBT. Finally, we discuss the validity of the QHA for the different anharmonic expressions and provide approximate design rules for controlling them.

**Table 1:**
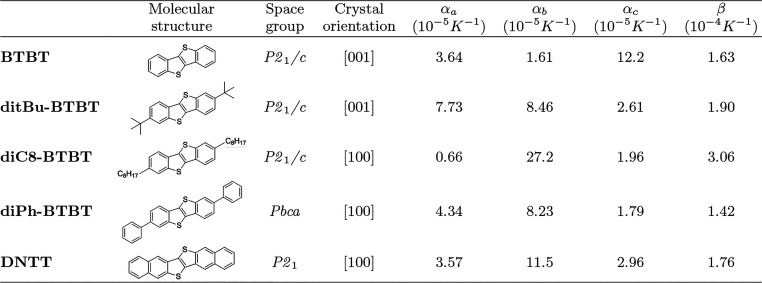
Molecular Structure, Space Group, Crystal Growth Orientation, and Uniaxial (α_*x*_) and Volumetric (β) Thermal Expansion Coefficients of the Examined Crystals

We note that, in this study, we probe only the Raman-active modes at the Γ-point, while the lattice dynamics also includes the Raman-inactive modes, acoustic modes, and the entire Brillouin zone, which can also significantly impact the macroscopic properties of organic solids. These characteristics of the lattice dynamics are beyond the scope of this work.

## Results

### Crystal Structure and Thermal Expansion Coefficients

Single crystals of BTBT, [2,7]-dioctyl-BTBT (**diC8-BTBT**), [2,7]di-*tert*-butyl-BTBT (**ditBu-BTBT**), [2,7]diphenyl-BTBT (**diPh-BTBT**), and dinaphtho[2,3-*b*:2,3-*f*]thieno[3,2-*b*]thiophene (**DNTT**) were prepared. Details regarding the synthesis, crystal growth, and characterization are provided in the Experimental Section and Supporting Information (SI) section S1. We confirmed the crystal structure and the high phase purity of the crystals by performing XRD measurements (see SI section S2). Table 1 presents their space group and crystal orientation. We also confirmed that most crystals exhibit a herringbone molecular packing and have the same space group as previously reported.^45−48^ One exception is diPh-BTBT, where the crystal structure of a new polymorph was obtained (see SI section S3 for detailed SC-XRD analysis).^49^

Next, we extract the thermal expansion coefficients of all five crystals by performing temperature-dependent SC-XRD measurements (see SI section S4). Table 1 shows the uniaxial (α_*x*_) and volumetric (β) thermal expansion coefficients for each crystal in its room temperature stable phase. As anticipated, the thermal expansion coefficients we obtain are relatively large (β ∼ 10^–4^ K^–1^) compared to those in inorganic solids (β ∼ 10^–6^–10^–5^ K^–1^),^50^ confirming their soft and anharmonic nature.

As mentioned in the Introduction, thermal expansion is only one possible expression of vibrational anharmonicity. Our results show that the chemical modifications do not significantly affect the thermal expansion coefficients as they are similar for all crystals.

### Temperature Evolution of the Raman-Active Lattice Vibrations

**Figure 1:**
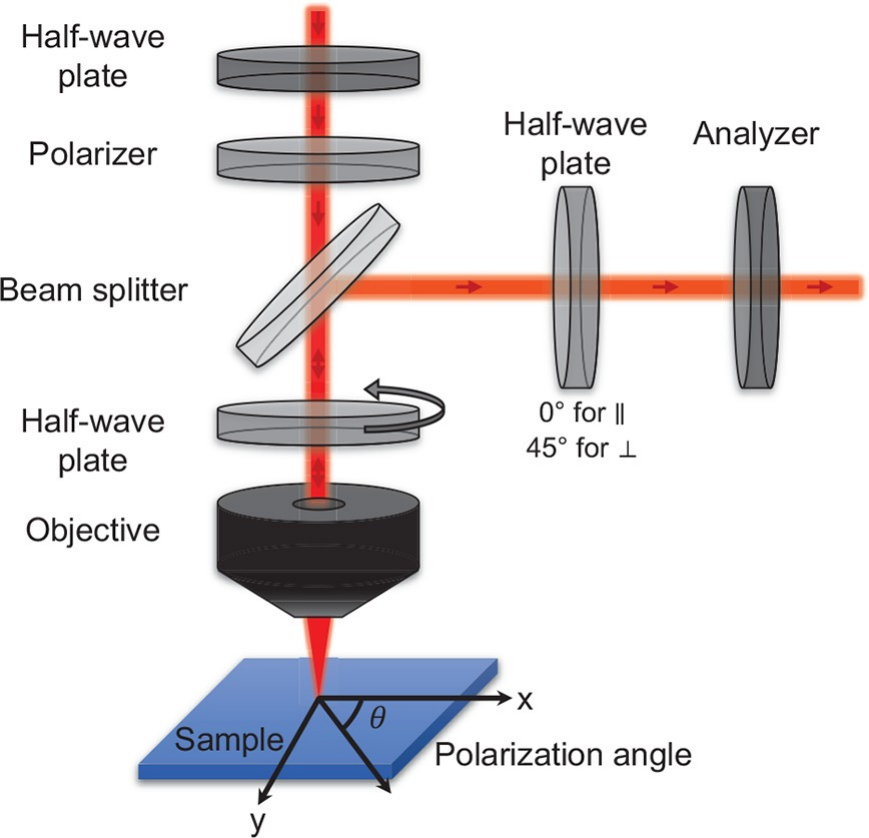
PO Raman measurement scheme. The first half-wave plate-polarizer combination ensures the linearity of the polarization and allows control over the laser intensity. After being passed through a beam splitter, a rotating half-wave plate determines the polarization angle of the laser with respect to the crystallographic axes of the crystal. The same half-wave plate rotates the polarization of the backscattered light to its initial polarization. Finally, we placed another half-wave plate-polarizer (analyzer) combination. As the analyzer is set parallel to the first polarizer, the half-wave plate angle with respect to the analyzer determines the measurement configuration, 0 or 45°, to measure the component of the signal, which is parallel or perpendicular to the incident laser polarization, respectively.

In the following sections, we explore the temperature evolution of the Raman-active lattice vibrations of all studied crystals via Raman scattering. Our study includes two types of data sets. The first is the unpolarized Raman spectrum as a function of temperature. From this, we extract the vibrational frequencies and the full width at half-maximum (fwhm) of each peak by fitting the data to the product of the Bose–Einstein distribution and a multi-damped Lorentz oscillator (see SI section S5 for more details). The second type of data is PO Raman. With this technique, we measure the change in Raman scattering intensity as a function of the angle between the linear polarization of the excitation laser and an arbitrary axis in the surface plane of the crystal (see Figure 1). More specifically, we probe the polarization-dependent Raman response within the 2D plane parallel to the surface. Since we measure in a backscattering configuration, the direction of the incident and scattered light is parallel to the crystal orientation (given in Table 1). Then we extract the vibrational symmetry of each mode according to its polarization-dependent integrated intensity (see SI section S6 for more details).

### BTBT

**Figure 2:**
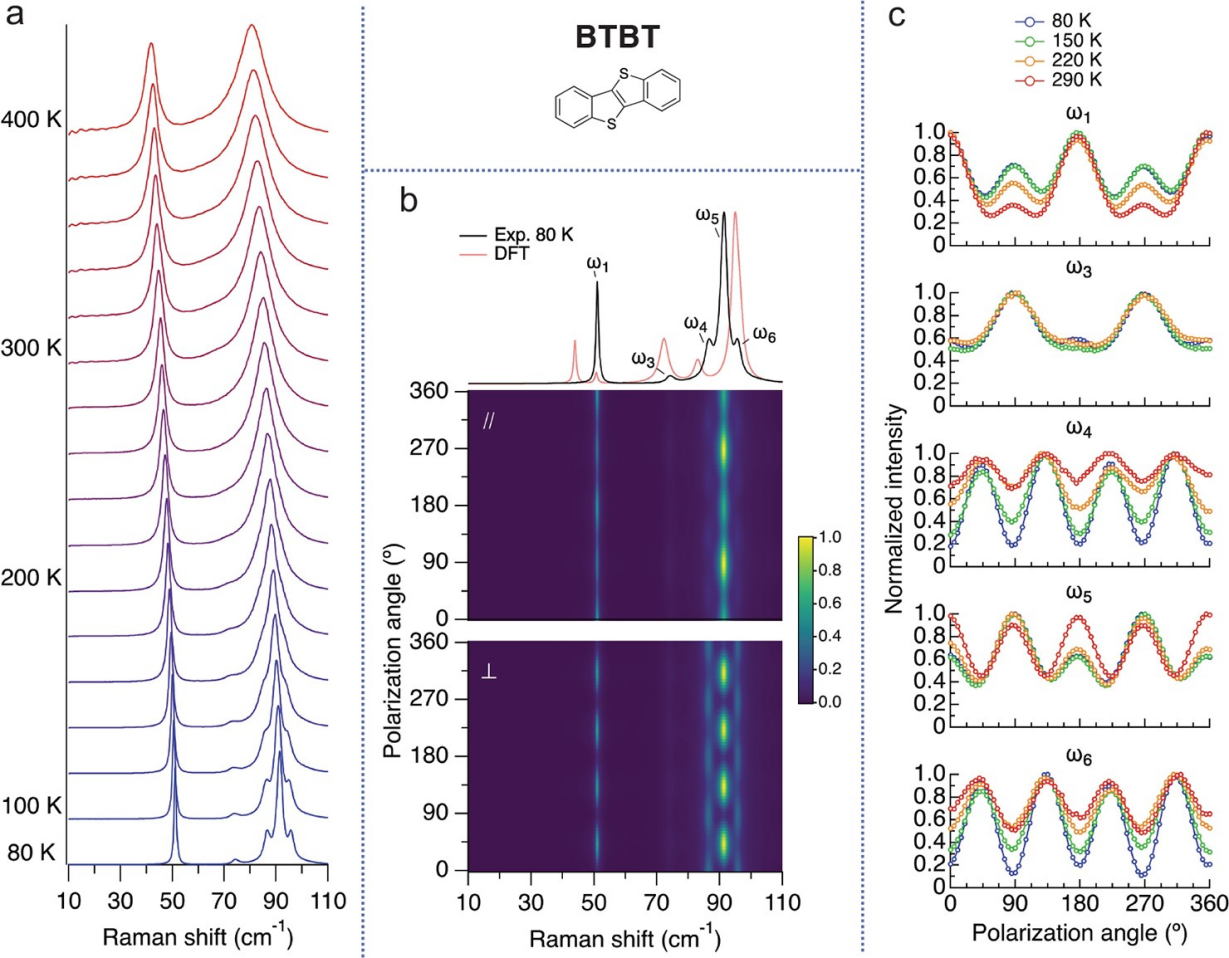
BTBT Raman measurements. (a) Temperature-dependent low-frequency Raman spectra. The spectra were normalized and shifted up for clarity. The temperature increment is 20 K. (b) Unpolarized Raman spectrum compared to the DFT-calculated spectrum (top panel), the PO dependence in the parallel (middle panel), and the perpendicular configuration (bottom panel) at 80 K. (c) Temperature dependence of the PO response for the low-frequency modes in the parallel configuration.

Figure 2a presents the results of the temperature-dependent low-frequency Raman measurements of BTBT. We observe a gradual decrease in frequency (i.e., ”red-shifting”) and broadening of the spectrum as temperature increases. From our fit, we learn that the vibrational frequencies and fwhm of the modes are linearly dependent on temperature (see SI section S7). This behavior is often observed^51−53^ since the temperature dependence of the vibrational frequencies and the fwhm values are proportional to the Bose–Einstein occupation factor (*n*). *n* is roughly linearly dependent on temperature when *k*_B_*T* is greater than *ℏ*ω (i.e., the vibrational energy).^53^ The change in vibrational frequencies with temperature is also captured by our DFT calculations performed at different temperatures using the QHA (i.e., accounting for the thermal expansion). These calculations indeed show a relatively rigid shift of all modes by 5–10 cm^–1^ in the explored temperature range (see SI section S8). Similar results were obtained in ref (30).

Next, we perform a PO measurement at 80 K (Figure 2b) and use it to assign a vibrational symmetry to each peak (see SI section S6). The results are shown for scattered light with parallel and perpendicular polarization with respect to the incident light. In the top panel of Figure 2b, we compare the unpolarized Raman spectrum to the DFT-calculated spectrum (using the experimental fwhm values). For all materials, we get a good agreement between the measured and DFT-calculated Raman spectra (see SI section S8 for more details). The calculated eigenvectors of the Raman-active modes for all crystals are attached to this publication as media files.

While the temperature evolution of the Raman spectrum of BTBT is benign, the temperature-dependent PO Raman measurements presented in Figure 2c reveal a more complex picture. To follow the evolution of the polarization-dependent response with temperature, we repeat the PO Raman measurements at higher temperatures (150, 220, and 290 K) and extract the fluctuation of the integrated intensity of each peak. The raw PO Raman data at higher temperatures of all five crystals are presented in SI section S9. Figure 2c shows the PO response temperature evolution in the parallel configuration of the lattice vibrations of BTBT (results of the perpendicular configuration of all crystals are provided in SI section S6; see Figure 1 for the definition of the parallel and the perpendicular configurations). The data are shown for the peaks labeled in Figure 2b (top panel). The integrated intensity of the unlabeled peak (ω_2_) was too low to reliably extract its PO Raman dependence.

In Figure 2c, we show that all peaks gradually change their PO Raman response as temperature increases, in both the parallel and the perpendicular configurations (see SI section S6 for the latter). This is a clear anharmonic effect, as in the harmonic picture, the PO response is expected to be temperature-independent (see SI section S10 for more details and an experimental example of the temperature independence of the PO response of silicon). To further investigate this anharmonic effect, we use a harmonic-based model to fit the PO Raman response of BTBT at 10 K where the anharmonic effects are minimal (see SI section S11). This allows the extraction of the Raman tensor of each mode, which in the harmonic picture is a second-rank tensor. However, to capture the temperature dependence of the PO Raman of BTBT, a fully anharmonic expansion is needed. This fully anharmonic model, presented by Cowley,^54^ gives rise to a fourth-rank Raman tensor^54^ and therefore new possible PO patterns. This fourth-rank Raman tensor is created by nonvanishing off-diagonal components in the autocorrelation function describing the dynamics, components that identically vanish in a purely harmonic system. The physical meaning of the nonvanishing off-diagonal components in the fourth-rank Raman tensor is specific coupling between normal modes (i.e., this model allows for mode mixing). In ref (55), we show how the fully anharmonic model is required to get a good fit to our data and extract the mixed irreducible representations participating in the scattering process. For each lattice mode, the degree of its PO Raman response temperature dependence depends on the coupling strength with other normal modes, which can be extracted by theory.^56^ This type of coupling is to be contrasted with nonspecific coupling to the heat bath, determined by the diagonal components of the self-energy of the Green’s function.^55^ These components create the frequency shift and finite lifetime (i.e., broadening of the peaks) of each individual mode.

As we will see below, the anharmonic expression of the temperature-dependent PO response is suppressed in the modified BTBTs. Taking these results into account with the results of ref (57), we see that the anharmonic expression in the PO Raman is uncorrelated with other anharmonic expressions, such as the red-shifting and broadening of the Raman peaks with temperature and thermal expansion. Importantly, the fact that the change in PO Raman with temperature is uncorrelated to the thermal expansion of the crystals indicates that the QHA framework cannot capture it.

### ditBu-BTBT and diC8-BTBT

**Figure 3:**
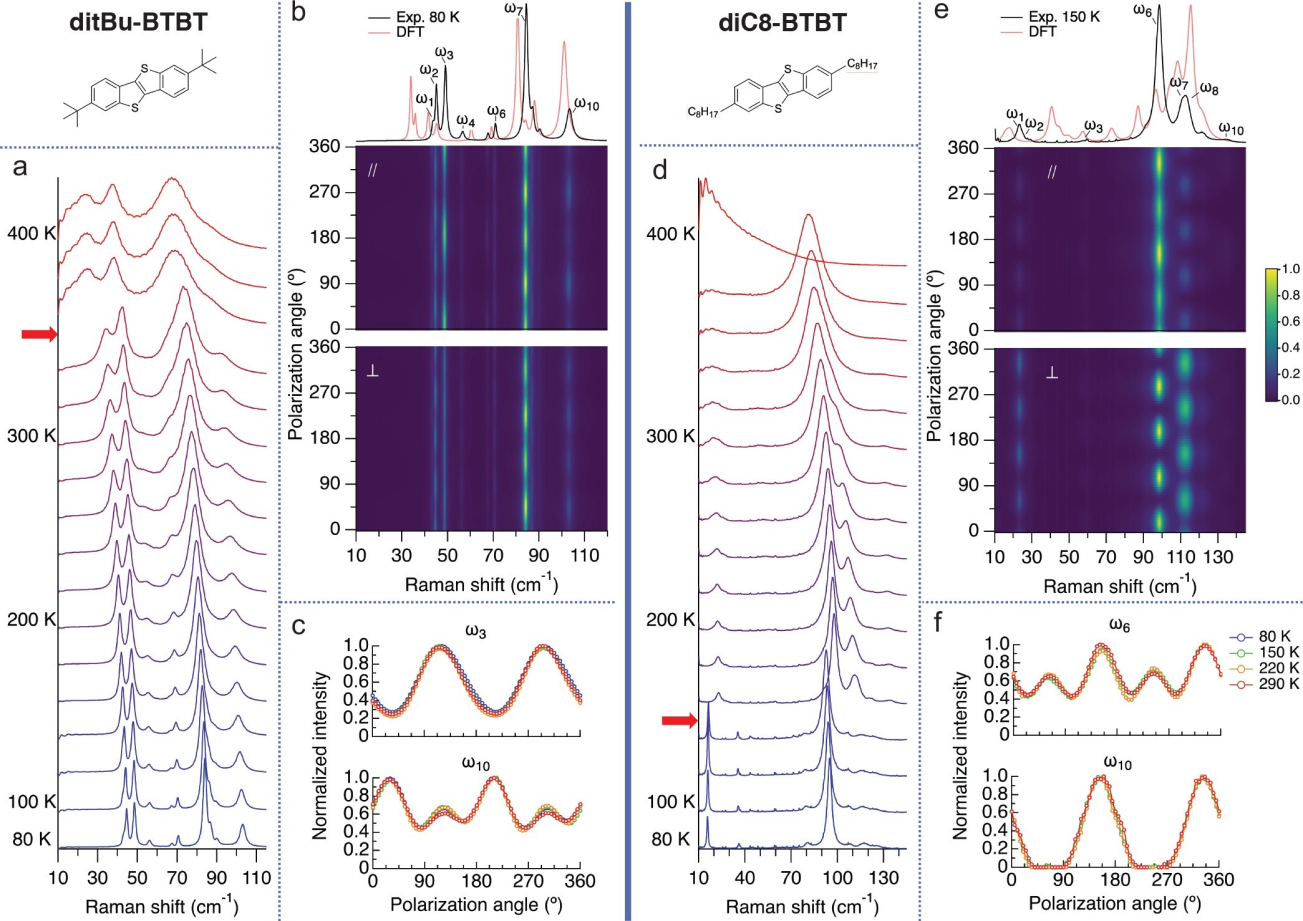
ditBu-BTBT and diC8-BTBT Raman measurements. (a,d) Temperature-dependent low-frequency Raman spectra. The spectra were normalized and shifted up for clarity. The temperature increment is 20 K. The solid-solid phase transitions are indicated by the red arrows. (b,e) Unpolarized Raman spectra compared to the DFT-calculated spectra (top panel), the PO dependence in the parallel (middle panel), and the perpendicular configuration (bottom panel) at 80 K for ditBu-BTBT and at 150 K for diC8-BTBT. (c,f) Temperature dependence of the PO response for the low-frequency modes in the parallel configuration.

ditBu-BTBT and diC8-BTBT are bundled together in this study because both have side chains with alkyl-based saturated bonds. Figure 3a,d presents the temperature-dependent Raman scattering results from 80 to 400 K for ditBu-BTBT and diC8-BTBT, respectively. These results show an abrupt phase transition in both crystals.

For ditBu-BTBT, we observe an abrupt red-shifting and broadening of the spectrum between 340 and 350 K. This is indicative of an order-to-disorder phase transition that was previously observed by some of us.^46^ The high-temperature phase of ditBu-BTBT is characterized by the rotation of the *tert*-butyl side chains, which introduces disorder to the system. However, the crystal structure is still monoclinic as in the low-temperature phase (space group *P*2_1_/*c*). The change in the Raman spectra at the phase transition reflects an abrupt weakening of the intermolecular interactions and shortening of the lattice vibrations’ lifetime due to the rotating side chains. We can also see that the overall shape of the Raman spectrum remains the same after this phase transition, indicating that the average crystal structure does not change. A more detailed analysis of the structural dynamics of this disordered phase is beyond the scope of this work and will be presented elsewhere.

For diC8-BTBT, we observe an abrupt change in the Raman spectrum between 140 and 150 K upon cooling and between 170 and 180 K upon heating, indicating a reversible phase transition between two polymorphs. To the best of our knowledge, this is the first time this phase transition has been reported. Therefore, we performed SC-XRD measurements to extract the average structure of the low-temperature phase (see SI section S12). The results indicate that the known monoclinic high-temperature phase transforms to triclinic in the low-temperature phase. The molecular packing also changes from herringbone to tilted and slipped cofacial packing (see SI section S12). Finally, between 380 and 390 K, the diC8-BTBT crystal melts, and the spectrum shown at 400 K is a typical Raman spectrum for liquids.^58,59^

Next, we measured the temperature evolution of the PO Raman response of both crystals. We use the same procedure performed for BTBT, where we extract the modulation of the integrated intensities of each peak to follow its evolution with temperature. Figure 3b,e presents the contour plots of the PO Raman data for ditBu-BTBT performed at 80 K and for diC8-BTBT at 150 K in the parallel and the perpendicular configurations along with their unpolarized Raman spectrum compared to the DFT-calculated spectrum. For diC8-BTBT, we start measuring the PO Raman response from 150 K since we are looking for its temperature evolution at the room temperature phase.

Figure 3c,f shows the temperature evolution of the PO response for representative peaks in the parallel configuration (see SI section S6 for the results of the rest of the labeled peaks). Contrary to the case of BTBT, where no phase transitions are observed but specific mode coupling emerges with temperature, we find that the PO response of all lattice vibrations in diC8-BTBT and ditBu-BTBT are unchanged. The suppression of the changing PO response with temperature in the modified crystals may be related to the previously reported smaller amplitude motion of their lattice vibrations with respect to BTBT.^9,44^ Suppressing the anharmonic PO response means that at all temperatures other than the phase transition temperature, the structural dynamics of the crystals can be captured by the QHA. We note that our last statement excludes the high-temperature, disordered phase of ditBu-BTBT that exhibits critical and overdamped phonons.

### diPh-BTBT and DNTT

**Figure 4:**
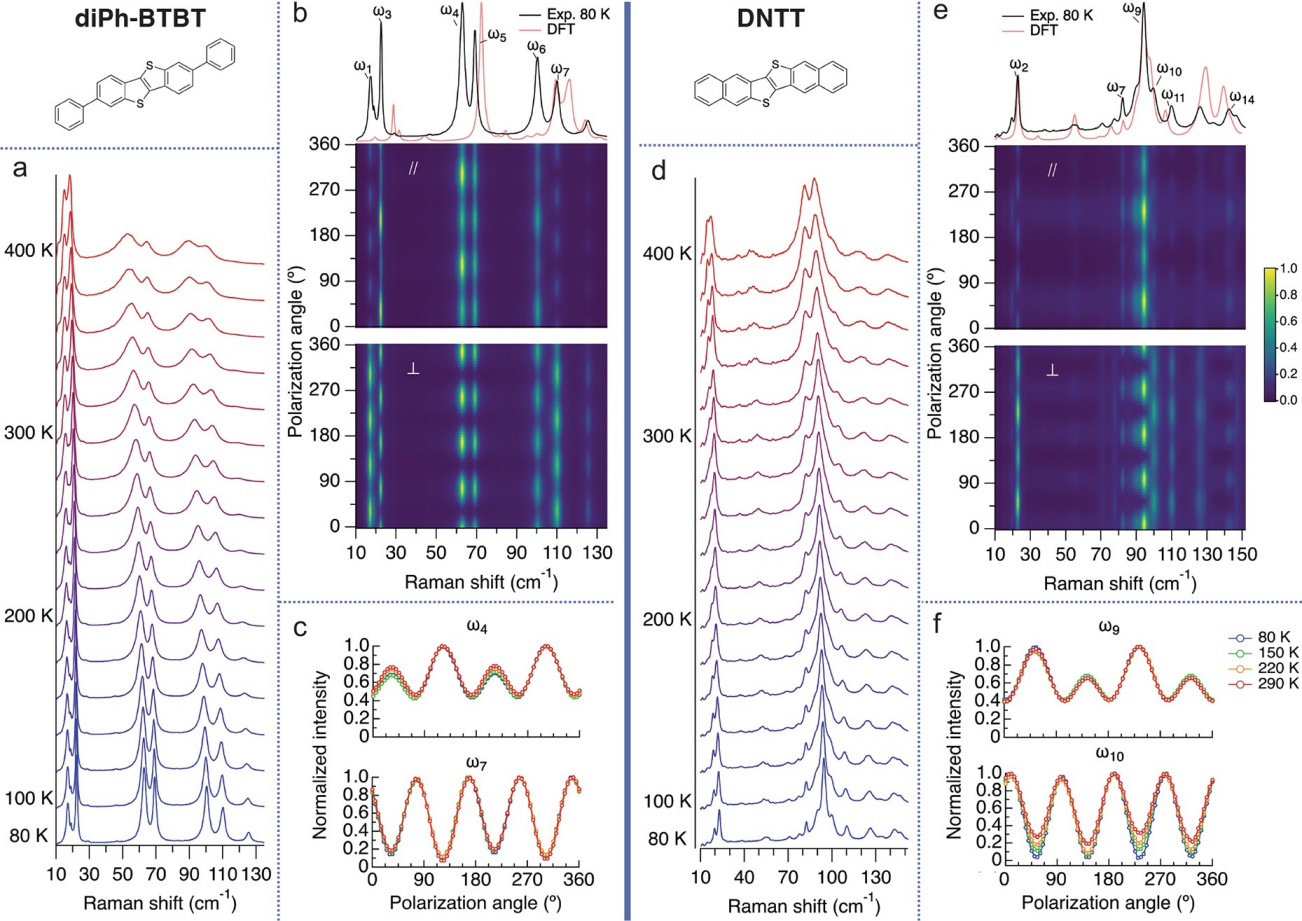
diPh-BTBT and DNTT Raman measurements. (a,d) Temperature-dependent low-frequency Raman. The spectra were normalized and shifted up for clarity. The temperature increment is 20 K. (b,e) Unpolarized Raman spectra compared to the DFT-calculated spectra (top panel), the PO dependence in the parallel (middle panel), and the perpendicular configuration (bottom panel) at 80 K. (c,f) Temperature dependence of the PO response for the low-frequency modes in the parallel configuration.

We repeat the same set of measurements described above for diPh-BTBT and DNTT. The main difference compared to ditBu-BTBT and diC8-BTBT is that the chemical modifications to the BTBT molecule include the addition of π-conjugated moieties, the phenyl groups for diPh-BTBT, and the fused benzene rings for DNTT. Figure 4a,d presents the Raman temperature dependence of the two crystals. Contrary to ditBu-BTBT and diC8-BTBT, diPh-BTBT and DNTT show no phase transitions in this temperature range. This could be due to the type of chemical modifications that are less prone to rotation and less flexible than those in ditBu-BTBT and diC8-BTBT. The temperature evolution of the peak positions and fwhm shows mostly a common linear red-shifting and broadening.

Another interesting finding is the temperature dependence of the lowest-frequency peaks (15–25 cm^–1^) of diPh-BTBT and DNTT. These peaks are relatively sharp (i.e., long vibrational lifetime) and are weakly dependent on temperature compared to the higher-frequency lattice vibrations. Thus, they are more harmonic with respect to these expressions of vibrational anharmonicity. However, lower-frequency modes are more susceptible to anharmonic effects as they are populated at room temperature (*kT* ≈ 200 cm^–1^) and are expected to have larger amplitude motion.^9^ Our results imply that the lowest frequency peaks of diPh-BTBT and DNTT have a potential energy surface that is more parabolic (i.e., harmonic) than the higher-frequency lattice vibrations. We attribute the harmonically shaped potential energy surface to the lack of available phonon decay paths (see SI section S7 for further discussion).

We complete our measurements with the PO Raman temperature evolution. Figure 4b,e presents the raw PO data at 80 K in the parallel and the perpendicular configurations along with their unpolarized spectrum compared to the DFT-calculated spectrum. Figure 4c,f presents the temperature evolution of the PO response of representative peaks (see SI section S6 for the results of the rest of the labeled peaks). In diPh-BTBT, the PO response is temperature-independent, similar to the previously described modified crystals. In DNTT, we do observe weak temperature dependence as specific peaks show some change with temperature of the PO response (represented by ω_10_ in Figure 4f). These results show that the π-conjugated modifications used here not only suppress the emergence of specific mode coupling with temperature found in BTBT but also have no solid–solid phase transition as was found in the alkyl-based modifications.

## Conclusions

Our results show various expressions of vibrational anharmonicity in the Raman-active lattice vibrations of BTBT and its derivatives. The main anharmonic effect in BTBT is the specific coupling of lattice vibrational modes to other modes in the system, a phenomenon that cannot be described within standard quasi-harmonic models. We find that chemical modifications to the BTBT molecule strongly suppress the emergence of this effect. For the alkyl side chain modifications (ditBu-BTBT and diC8-BTBT), solid–solid phase transitions emerge while π-conjugated chemical modifications (diPh-BTBT and DNTT) suppress all forms of strong anharmonic behavior.

## Experimental Section

### Crystal Growth

BTBT single crystals were grown by leaving a saturated BTBT solution in chloroform in open air at room temperature until the chloroform was completely evaporated. The same method was applied to obtain diC8-BTBT single crystals from a saturated solution in heptane in a glass vial closed with a plastic cap. ditBu-BTBT, diPh-BTBT, and DNTT single crystals were grown by thermal sublimation in a Severn Thermal Solutions *TF*50/7.5/3*Z*/*F* furnace at 315 °C with a temperature gradient of −2 K/cm under an argon flow of 0.5 mL/min, at 340 °C with a temperature gradient of −4 K/cm under an argon flow of 0.5 mL/min, and at 400 °C with a temperature gradient of −4 K/cm under an argon flow of 0.5 mL/min, respectively.

### Temperature-Dependent PO Raman

A custom-built Raman system was used to conduct the Raman measurements. The system included a 785 nm Toptica diode laser with an intensity of around 30 mW on the sample. To control the polarization of the incident and scattered light for the polarization-dependent measurements (steps of 5°), rotating half-wave plates and a polarizer–analyzer combination were used. The system included a 50× objective. Notch filters are included in the system to allow access to the low-frequency region (>10 cm^–1^) and simultaneous acquisition of the Stokes and anti-Stokes signals. The system is based on a 1 m long Horiba FHR-1000 dispersive spectrometer with a 1800 mm^–1^ grating. The spectral resolution was approximately 0.15 cm^–1^. Section S13 presents the minimal system response of our system by showing the PO response of chloroform, which is polarization-independent, as expected from a liquid sample. The temperature was set and controlled by a Janis cryostat ST-500 and a temperature controller by Lakeshore model 335. Due to its abrupt phase transition, the diC8-BTBT crystal was measured free-standing inside a custom-built encapsulated cell with a helium environment placed inside the cryostat.

### Temperature-Dependent Single-Crystal XRD

Cell dimension measurements were performed on a Rigaku Xtalab PRO dual-source diffractometer equipped with Dectris Pilatus 200 K detector and microfocus using Cu Kα (λ = 1.54184 Å). All crystals from all samples (diC8-BTBT, DiPh-BTBT, DNTT, BTBT, and ditBu-BTBT) were very thin plates. The same data collection strategy was applied to all different temperature measurements of the same crystal. Variant temperature measurement is a module of CrysAlis PRO. The measurement protocol consists of mounting a single crystal with a minimal amount of Paratone oil at 300 K. A short data collection strategy was determined to establish the most accurate cell parameters in the shortest possible time. The same crystal was measured at five different descending temperatures of 300, 250, 200, 150, and 100 K. The cooling rate was 1 K/min with a 5 min thermalization time. Before each measurement, a crystal video was taken to visually check the crystal’s position and appearance. All measurements were completed. For diC8-BTBT, at 150 K, the crystal is damaged but still diffracting (see SI section S12). However, at 100 K, there was no diffraction at all. The cell parameters of diC8-BTBT at 100 K are contributed from another crystal flash cooled and structure fully refined and reported in SI section S12. Data from each temperature measurement were integrated, scaled, and solved by direct methods with SHELXT.^60^ A large section of the Ewald sphere was collected to establish the cell parameters. We assume that if the accumulated partial data were enough to solve the structure, it would also give accurate enough cell parameters. The cell parameters were extracted from the unfinished CIF of the partial data set. For the experimental details regarding the powder XRD measurements and SC-XRD measurement of the diPh-BTBT crystal, see SI sections S2 and S3, respectively.

### DFT Calculations

Solid-state DFT simulations were performed using the fully periodic CRYSTAL17 software package.^61,62^ All structures underwent geometry optimization, and the calculations were initiated using the experimental atomic positions and lattice vectors retrieved from Cambridge Crystallographic Data Centre (CCDC) or from measurements described in the previous section, which were also the reference for QHA calculations lattice vector data for each temperature value. Prior to any vibrational analyses, all atoms were allowed to fully relax with no constraints other than the space group symmetry of the solid and the lattice vectors. Frequency calculations were executed using the optimized coordinates to yield the vibrational modes and Raman intensities. Eigenvalues and eigenvectors were calculated numerically through the harmonic approximation,^63^ and Raman intensities were calculated from the dipole moment derivatives, which were determined using the Berry phase method.^64^ Reciprocal space sampling was performed using the Monkhorst–Pack scheme, with a k-point mesh in the first Brillouin zone (program keyword SHRINK: X X X). The tolerances for Coulomb and exchange integral cutoffs were set to Δ*E* < 10^–8^ Hartree (program keyword TOLINTEG: 8 8 8 8 16). The energy convergence criterion for geometric optimizations was set to Δ*E* < 10^–12^ Hartree (program keyword TOLDEE: 12). The energy convergence criterion for frequency calculations was likewise set to Δ*E* < 10^–12^ Hartree. The Pople 6-31G* basis set^65,66^ was utilized for all calculations. The generalized gradient approximation (GGA) class functional Perdew–Burke–Ernzerhof (PBE)^67^ was used for all calculations. London dispersion forces were accounted for using the Grimme DFT-D3 correction.^68^

## Supporting Information Available

Optical photos of the crystals, X-ray diffraction results, raw temperature and polarization-dependent Raman results and analysis description, DFT results, and mode assignment (PDF)
