# Supplementary material for: Chemical Modifications Suppress Anharmonic Effects in the Lattice Dynamics of Organic Semiconductors
Source: ACS Mater Au. 2022 Jul 5;2(6):699–708. doi: 10.1021/acsmaterialsau.2c00020 (PMC9650719; doi:10.1021/acsmaterialsau.2c00020)
Supplement: Supplementary file 1 — mg2c00020_si_001.pdf [file mg2c00020_si_001.pdf]

Supporting Information

Chemical modifications suppress anharmonic effects in the lattice dynamics of organic semiconductors

Maor Asher,[1] Rémy Jouclas,[2] Marco Bardini,[3] Yael Diskin-Posner,[4] Nitzan Kahn,[1] Roman Korobko,[1] Alan R. Kennedy,[5] Lygia Silva de Moraes,[2] Guillaume Schweicher,[2] Jie Liu[2], David Beljonne,[3] Yves Geerts,[2,6] and Omer Yaffe[1]*

[1]Department of Chemical and Biological Physics, Weizmann Institute of Science, Rehovot 76100, Israel.

[2]Laboratoire de Chimie des Polymères, Université Libre de Bruxelles (ULB), 1050 Brussels, Belgium

[3]Laboratory for Chemistry of Novel Materials, University of Mons, 7000 Mons, Belgium

[4]Chemical Research Support, Weizmann Institute of Science, Rehovot [5]76100, Israel

Department of Pure and Applied Chemistry, University of Strathclyde, G1 1XL Glasgow, UK

[6]International Solvay Institutes for Physics and Chemistry, 1050 Brussels, Belgium

*omer.yaffe@weizmann.ac.il

S1 Optical photos of the crystal

Figure S1 presents an optical photo of typical crystals which were grown according to the growth methods given in the manuscript. The crystals have a platelet shape, a few millimeters square in size.

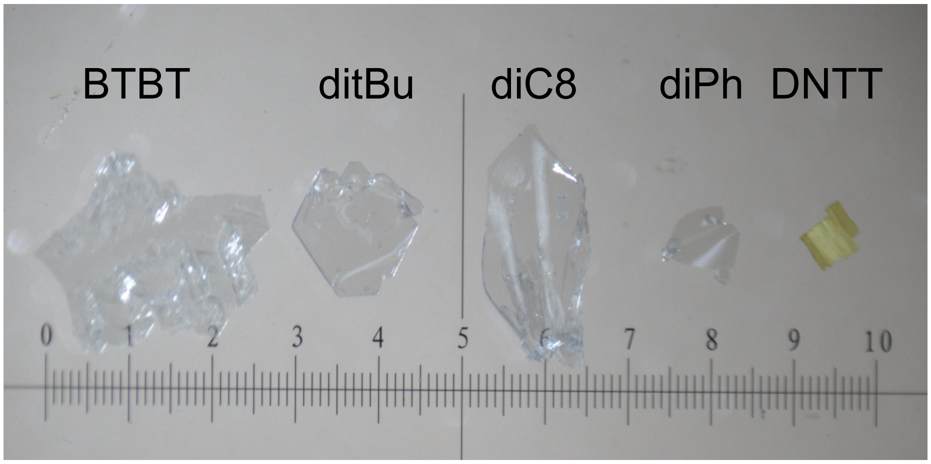

Figure S1: An optical photo of the crystals used in this study: BTBT, ditBu-BTBT, diC8-BTBT, diPh-BTBT, and DNTT. The scale is in millimeters.

S2 Powder X-ray diffraction measurements

Preferred orientation and phase purity of BTBT, ditBu-BTBT, diC8-BTBT, DNTT, and diPh-BTBT were analyzed using powder X-ray diffraction. All the experiments were performed at room temperature. Compounds were finely ground using a mortar and pestle for phase confirmation measurement. Single crystals were mounted over the sample holder for preferential orientation analysis. For BTBT and ditBu-BTBT, the measurements were conducted on a Panalytical Empyrean diffractometer using Cu–Kα radiation (λ =1.54178 Å). The diffractometer was set up with reflection-transmission spinner 3.0 configuration, and patterns were collected with 2θ range between 5.0 and 30.0°, steps of 0.1°, time per step of 2.5 s, and rotation of 1 r/s. For diC8-BTBT, DNTT and diPh-BTBT, the measurements were performed on a Rigaku Ultima IV diffractometer with Cu–Kα radiation (λ =1.54178 Å). Diffraction patterns covering the range of 2θ from 2° (for diC8-BTBT) and 4° (for DNTT and diPh-BTBT) were collected with an angular resolution of 0.02° per step and a scan speed of 6 °/min, using the 2θ / omega reflection geometry. Calculated patterns were obtained from known crystal structures of BTBT [1–3], ditBu-BTBT [4, 5], diC8-BTBT [6], DNTT [7], and diPh-BTBT [8] using Powder Pattern tool on Mercury software [9, 10]. The crystalline phase of BTBT, ditBu-BTBT, diC8-BTBT, and DNTT were confirmed to have the same phase of the known crystal structures with CSD Refcode PODKEA02 [3] (BTBT), KUDFAS01 [5] (ditBu-BTBT), YOKBIK [6] (diC8-BTBT), and NICLAN [7] (DNTT) as it can be observed in Figure S2 (experimental diffraction pattern in red and calculated diffraction patterns in black). However, the diffraction peaks of diPh-BTBT are associated with a new form obtained by the sublimation method in the crystal growth section rather than the known form of diPh-BTBT [UVAHEF] [8]. The single-crystal structure of this new form was solved and deposited in CCDC with the CCDC No. 2103574 (see section S3). The XRD patterns can be observed in Figure S2. Single crystals of compounds BTBT, ditBu-BTBT with highly (100) preferred orientation crystallized along [001] direction; diC8-BTBT, diPh-BTBT and DNTT with highly (001) preferred orientation crystallized along [100] direction.

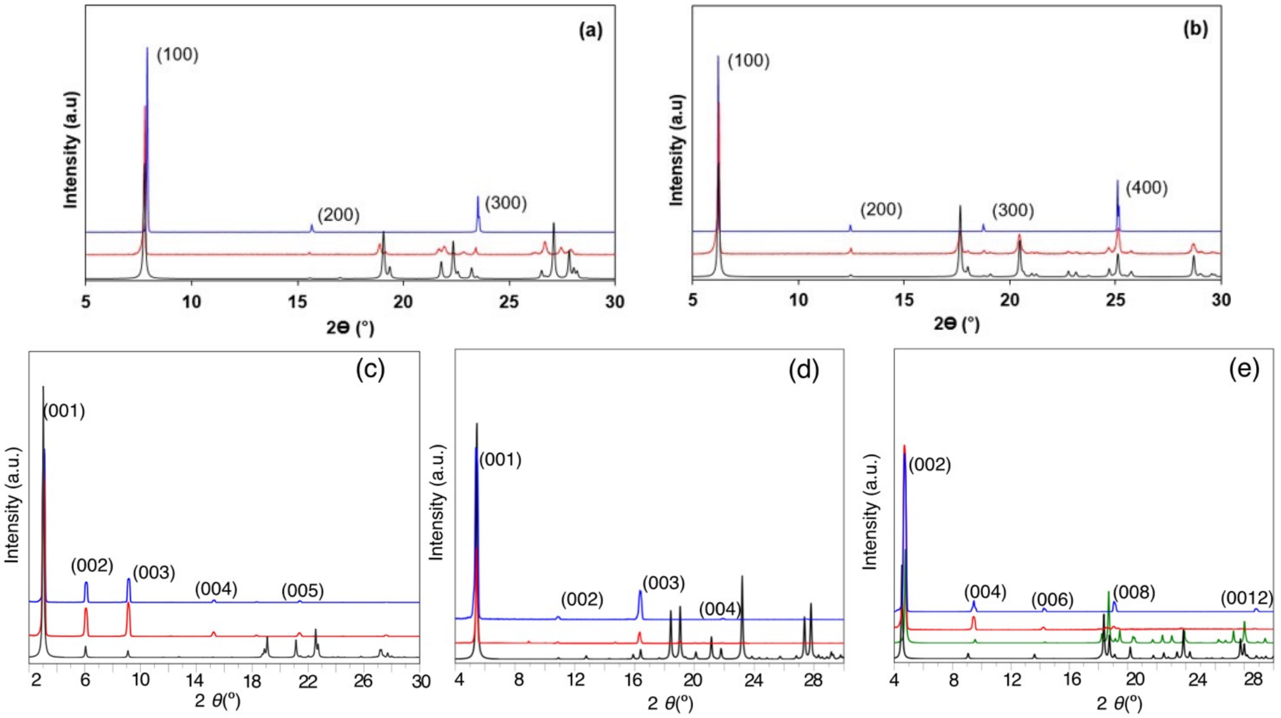

Figure S2: X-Ray diffraction patterns calculated from the known crystal structures (in black), the new form of diPh-BTBT (in green), obtained experimentally from powders (in red) and obtained experimentally from single crystals (in blue) of (a) BTBT. (b) ditBu-BTBT. (c) diC8-BTBT. (d) DNTT and (e) diPh-BTBT.

S3 Single-crystal XRD of diPh-BTBT

Single crystal X-ray diffraction data for diPh-BTBT were measured with an Oxford Diffraction Gemini S instrument using Cu–Kα (λ =1.54184 Å) radiation. Data collection and processing used CrysalisPro software [11]. The structure was refined to convergence on F^2 using all independent reflections and the program SHELXL-2018 as implemented within WinGX[12, 13]. The non-hydrogen atoms were refined anisotropically, and hydrogen atoms were placed in idealized positions and refined in riding modes. Selected crystallographic data and refinement parameters are presented in Table S1 and a structural figure showing displacement ellipsoids is given in Figure S3. CCDC deposition number 2103574 contains the full supplementary crystallographic data for this paper in CIF format. These data are provided free of charge by the joint Cambridge Crystallographic Data Centre and Fachinformationszentrum Karlsruhe Access Structures service www.ccdc.cam.ac.uk/structures.

Table S1: Single-crystal XRD of diPh-BTBT - selected crystallographic data and refinement parameters.

**Compound**	**diPh-BTBT**
**CCDC**	2103574
**Formula**	$C_{26}H_{16}S_2$
**Form. Wt.**	392.51
**Space Group**	*Pbca*
**Crystal System**	Orthorhombic
**Temp. (K)**	123(2)
**a (Å)**	6.1487(2)
**b (Å)**	8.1247(2)
**c (Å)**	37.0329(9)
Z	4
**Z'**	0.5
**Measured Reflections**	5632
2θ**max** (°)	145.836
R_{int}	0.0961
**Observed Reflections [**$I > 2\sigma I$**]**	1597
**No. Parameters**	127
**S**	1.036
**R [on F, obs refs only]**	0.0744
ωR **[on F2, all data]**	0.2044
L**argest diff. peak /hole (**eÅ^{-3}**)**	0.842/-0.535

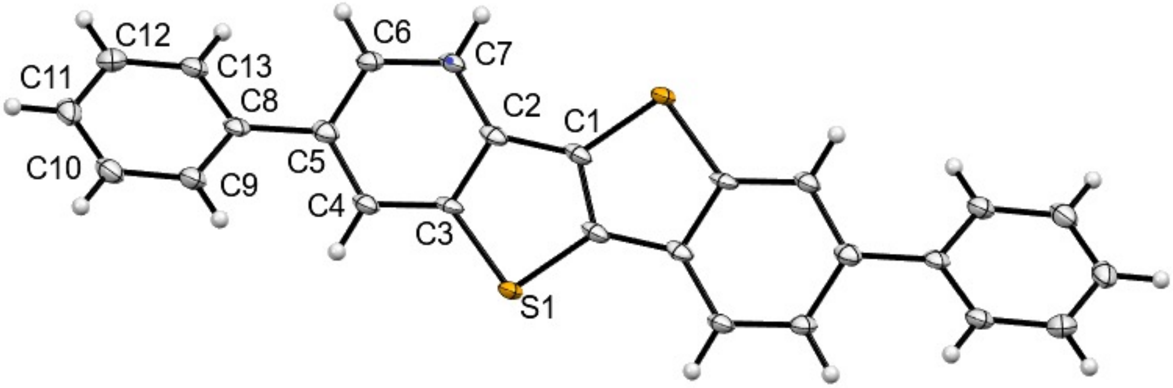

Figure S3: ORTEP representation of the structure of diPh-BTBT [12]. The molecule has a crystallographic centre of symmetry, with the non-labeled atoms generated by the operation 2-x, 1-y, -z. The non-H atoms are shown as 50% probability ellipsoids and the H atoms are shown as small spheres of arbitrary size.

S4 Temperature dependent single-crystal X-ray diffraction measurements

We perform single-crystal XRD measurements on BTBT, ditBu-BTBT, diC8-BTBT, diPh-BTBT, and DNTT between 100-300 K at increments of 50 K to extract their thermal expansion coefficients. The growth procedures of the five crystals are mentioned in the main text. Tables S2-S6 present the results for the unit cell parameters at every temperature for each material. The thermal expansion coefficients presented in the main test were calculated by dividing the slope of the lattice parameter with respect to the temperature with its intercept at 0 K. For BTBT, similar results for the unit cell volume were obtained in Ref. [14]. For diC8-BTBT, the coefficients were calculated without the measurement at 100K due to the phase transition.

Table S2: Single-crystal XRD measurements of BTBT.

Temperature (K)	a (Å)	b (Å)	c (Å)	α (°)	β (°)	γ (°)	V (Å^3)
300	11.8892(3)	5.8910(2)	8.1111(3)	90	106.501(3)	90	544.70(3)
250	11.8656(3)	5.88510(10)	8.0603(2)	90	106.351(2)	90	540.09(2)
200	11.8427(3)	5.87980(10)	8.0099(2)	90	106.212(2)	90	535.57(2)
150	11.8221(3)	5.87570(10)	7.9629(2)	90	106.081(2)	90	531.48(2)
100	11.8040(3)	5.87210(10)	7.9215(2)	90	105.973(2)	90	527.87(2)

Table S3: Single-crystal XRD measurements of ditBu-BTBT

Temperature (K)	a (Å)	b (Å)	c (Å)	α (°)	β (°)	γ (°)	V (Å^3)
300	14.1896(5)	6.1575(2)	10.6486(3)	90	92.355(3)	90	929.61(5)
250	14.1225(7)	6.1221(2)	10.6345(4)	90	92.038(4)	90	918.87(6)
200	14.0690(8)	6.0956(3)	10.6183(5)	90	91.773(5)	90	910.18(8)
150	14.0177(10)	6.0710(4)	10.6050(5)	90	91.549(5)	90	902.17(10)
100	13.9742(9)	6.0562(3)	10.5944(5)	90	91.397(5)	90	896.34(8)

Table S4: Single-crystal XRD measurements of diC8-BTBT

Temperature (K)	a (Å)	b (Å)	c (Å)	α (°)	β (°)	γ (°)	V (Å^3)
300	29.1177(7)	7.8868(2)	5.91580(10)	90	92.119(2)	90	1357.61(5)
250	29.1155(8)	7.7503(2)	5.9018(2)	90	92.507(2)	90	1330.49(7)
200	29.1116(8)	7.6593(2)	5.8984(2)	90	92.651(3)	90	1313.79(7)
150	29.0868(14)	7.5867(5)	5.8977(3)	90	92.753(4)	90	1299.96(13)
100	8.1001(4)	5.6595(3)	27.2931(11)	93.776(4)	93.815(7)	93.932(4)	1243.76(10)

Table S5: Single-crystal XRD measurements of diPh-BTBT.

Temperature (K)	a (Å)	b (Å)	c (Å)	α (°)	β (°)	γ (°)	V (Å^3)
300	6.1999(3)	8.2131(4)	37.1818(18)	90	90	90	1893.31(16)
250	6.1915(2)	8.1873(5)	37.171(2)	90	90	90	1884.28(16)
200	6.1800(3)	8.1490(5)	37.118(2)	90	90	90	1869.28(18)
150	6.1648(2)	8.1120(3)	37.0773(16)	90	90	90	1854.19(12)
100	6.1523(4)	8.0858(5)	37.063(3)	90	90	90	1843.7(2)

Table S6: Single-crystal XRD measurements of DNTT.

Temperature (K)	a (Å)	b (Å)	c (Å)	α (°)	β (°)	γ (°)	V (Å^3)
300	6.2293(7)	7.7154(10)	16.261(3)	90	92.276(12)	90	780.91(18)
250	6.2283(8)	7.6630(12)	16.225(3)	90	92.301(13)	90	773.8(2)
200	6.2078(13)	7.6153(16)	16.193(3)	90	92.198(17)	90	764.9(3)
150	6.1994(8)	7.5760(12)	16.1768(19)	90	92.035(10)	90	759.29(18)
100	6.1993(11)	7.5442(16)	16.166(3)	90	91.958(16)	90	755.6(3)

S5 Raman spectra fitting

We fit the measured Stokes-shift Raman spectra with the product of the Bose-Einstein distribution and a multi-damped Lorentz oscillator line shape,

$$I_{Raman}(\omega) = \left(\frac{1}{e^{\frac{\hbar\omega}{k_B T}} - 1} + 1 \right) \sum_i \frac{c_i |\omega| \Gamma_i^3}{\omega^2 \Gamma_i^2 + (\omega^2 - \omega_i^2)^2} \quad (1)$$

Where $\omega_{0,i}$, c_i and Γ_i are the position, intensity, and width of each peak, respectively, ω is the measured frequency (Raman shift), T is the temperature, $\hbar$ is the Planck constant and k_B is the Boltzmann constant. The Lorentz in Equation 1 is a variation of the Lorentz oscillator where c is the max value of the peak.

S6 Temperature dependent polarization-orientation (PO) Raman vibrational response

Figures S4 and S5 present the temperature evolution of the lattice vibrations with temperature for each crystal. The specific vibrations shown in these figures are presented in the parallel and perpendicular configurations, respectively. The results show similar observations to those presented in the main text for representative peaks in the parallel configuration where the PO dependence of BTBT shows significant changes as the temperature increases. In contrast, for the rest, the PO dependence is maintained. Figure S6 presents the temperature evolution of the PO response of the rest of the lattice vibrations of DNTT. They show similar behavior to the vibrations presented in the main text, where only specific modes show a gradual change of the PO response with temperature. By analyzing the eigenvectors of each mode obtained from the DFT calculations, we see no correlation between the type of motion and the strength of the PO Raman anharmonic response.

From the PO response, we also extract the vibrational symmetry of each mode. From factor group analysis, we know that the Raman tensor of A_g modes includes only diagonal components while the Raman tensor of B_g modes includes only off-diagonal components (see section S11, the rest of the crystals have similar results). The expressions we obtain from Eq. 2 using these Raman tensors show that in parallel configuration, the polarization dependence of an A_g mode has a $sin^4\theta$ and/or $cos^4\theta$ dependency (where θ is the polarization angle) and a B_g mode has a $sin^2\theta cos^2\theta$ dependency. This allows us to distinguish between A_g and B_g modes since while the PO pattern of B_g modes must have four peaks (for example see the PO pattern of ω_1 of diPh-BTBT in Figure S4), the PO pattern of A_g modes can also have two peaks (for example see the PO pattern of ω_3 of ditBu-BTBT in Figure S4). In addition, the maxima points in the PO pattern of the A_g modes will be in a 45 degrees shift to the maxima points in the PO pattern of the B_g modes.

For example we show in Figure S7 the Raman spectra of ditBu-BTBT at 80 K in three polarization angles. Two of these angles are the maximum and minimum intensity of a clear A_g mode (ω_3 at 105 and 195 degrees) and the third is in 45 degrees shift (150 degrees). The maxima points in the PO pattern of A_g modes usually occur when the polarization is parallel to a crystallographic axis. The A_g modes are those which have their highest intensity when the po-

larization angle is aligned with a crystallographic axis, while the B_g modes are those which have their highest intensity when the polarization angle is aligned in-between the crystallographic axes. We performed the same procedure to assign the vibrational symmetries for the rest of the crystals. The results are presented in section S8.

According to Eq. 2, in some cases, the minima points in the PO response are expected to go down to zero, for instance, the PO response of the B_g modes in parallel and perpendicular configurations. However, for many organic crystals, the intensity never drops to zero. To settle this discrepancy, we suggest and refute different possible sources. From sections S10 and S13, we know that our system response is minimal. Also, the birefringent nature of these crystals is taken into account in the model. The most probable sources are crystal imperfections and tilting (we assume the crystals are perpendicular to the laser beam. Importantly, none of these sources can explain the reversible and temperature-dependent PO Raman response in BTBT (which is performed on the same spot of the crystal).

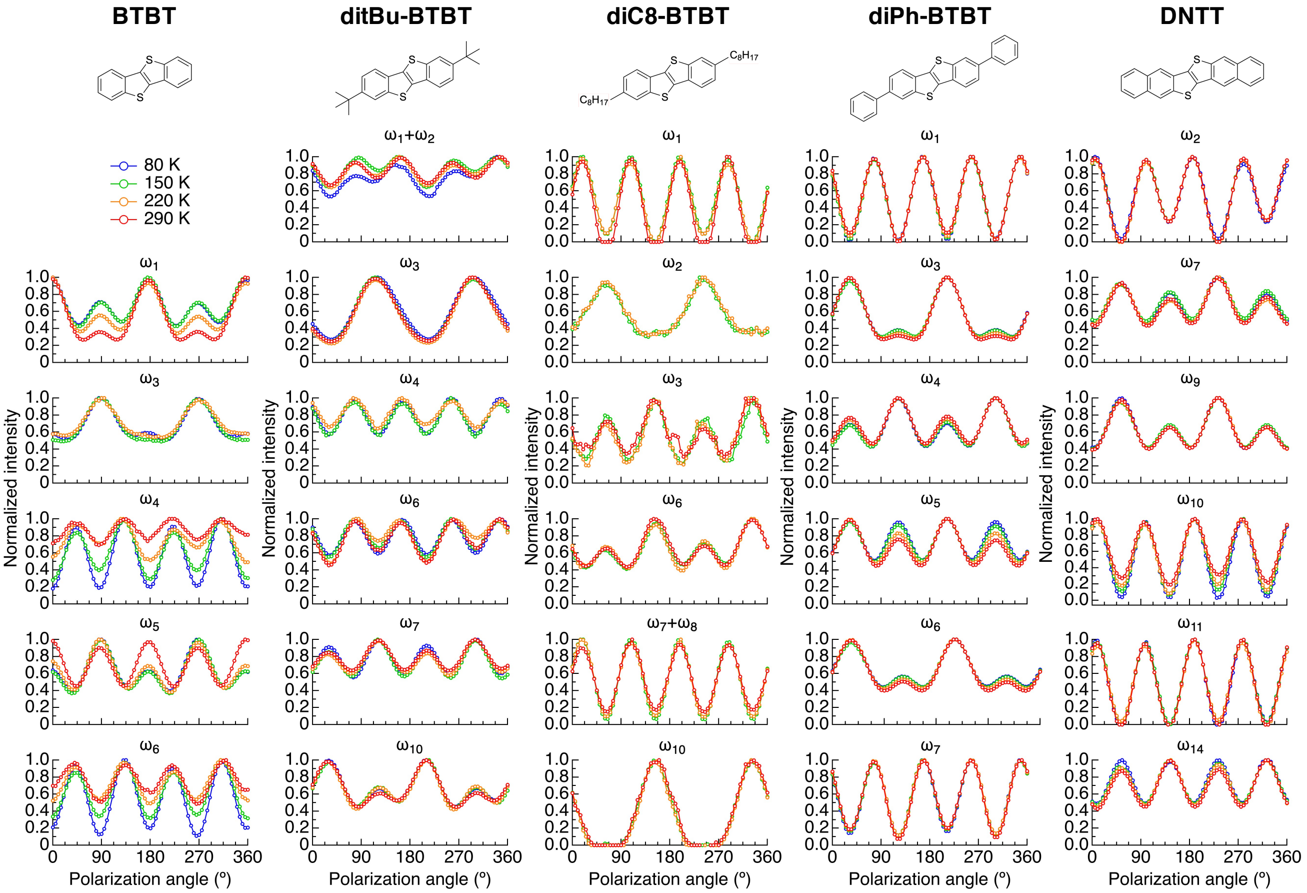

Figure S4: The temperature evolution of the PO dependence for the low-frequency peaks of BTBT, ditBu-BTBT, diC8-BTBT, diPh-BTBT, and DNTT in parallel configuration. The intensities were normalized for each mode at each temperature.

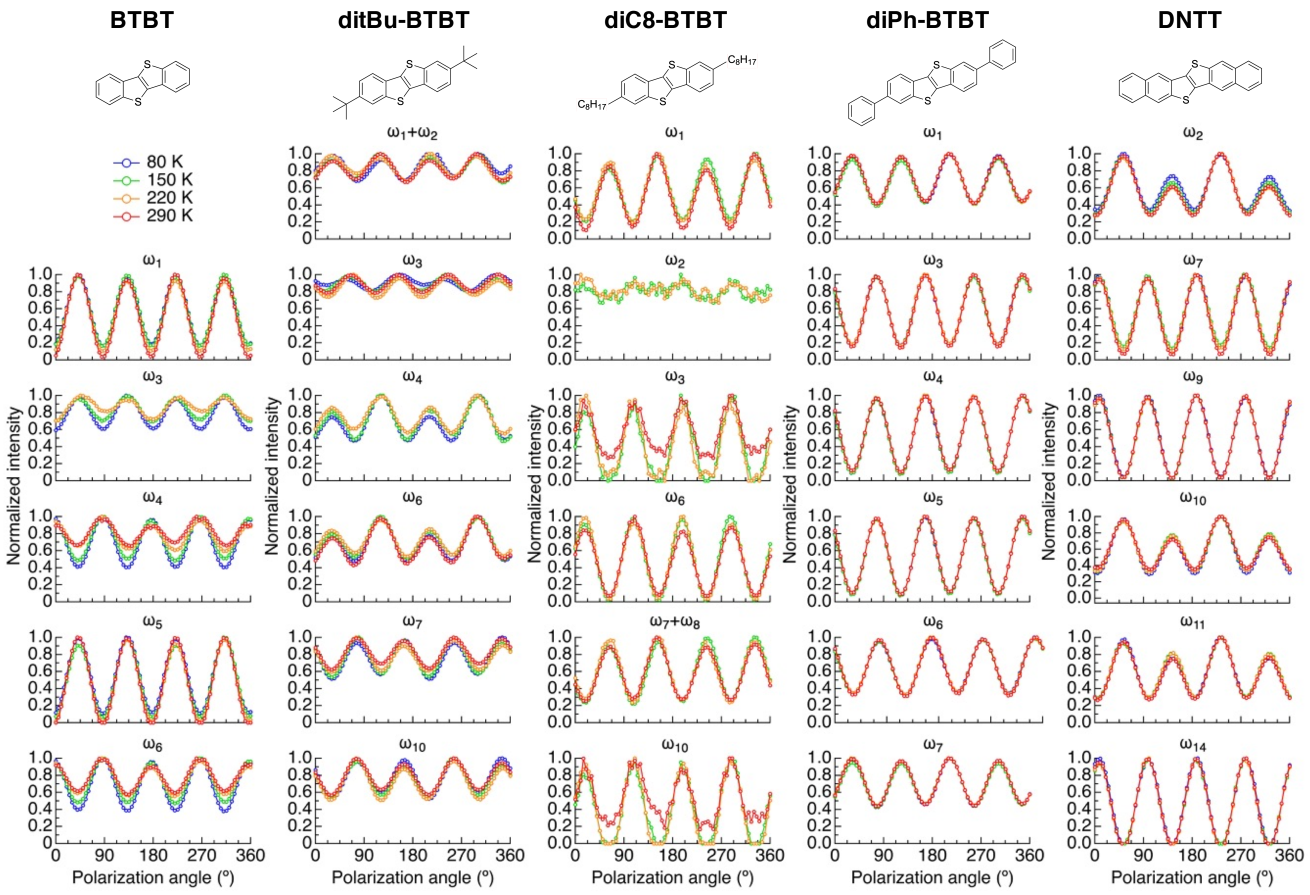

Figure S5: The temperature evolution of the PO dependence for the low-frequency peaks of BTBT, ditBu-BTBT, diC8-BTBT, diPh-BTBT, and DNTT in perpendicular configuration. The intensities were normalized for each mode at each temperature.

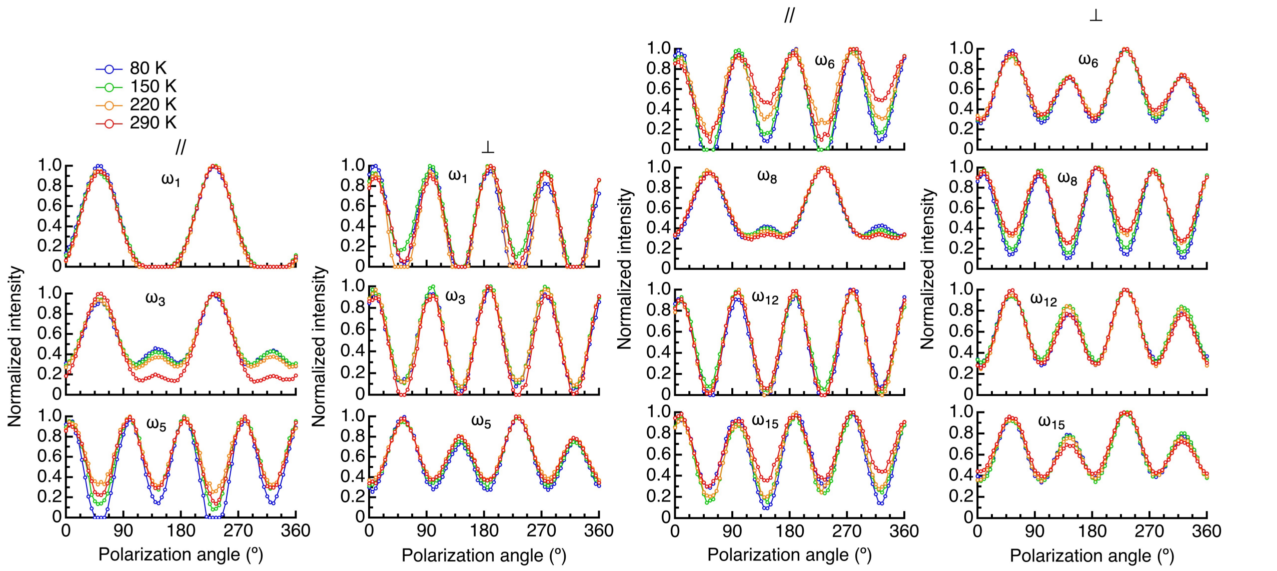

Figure S6: The temperature evolution of the PO dependence for the low-frequency peaks of DNTT in parallel and perpendicular configuration. The intensities were normalized for each mode at each temperature.

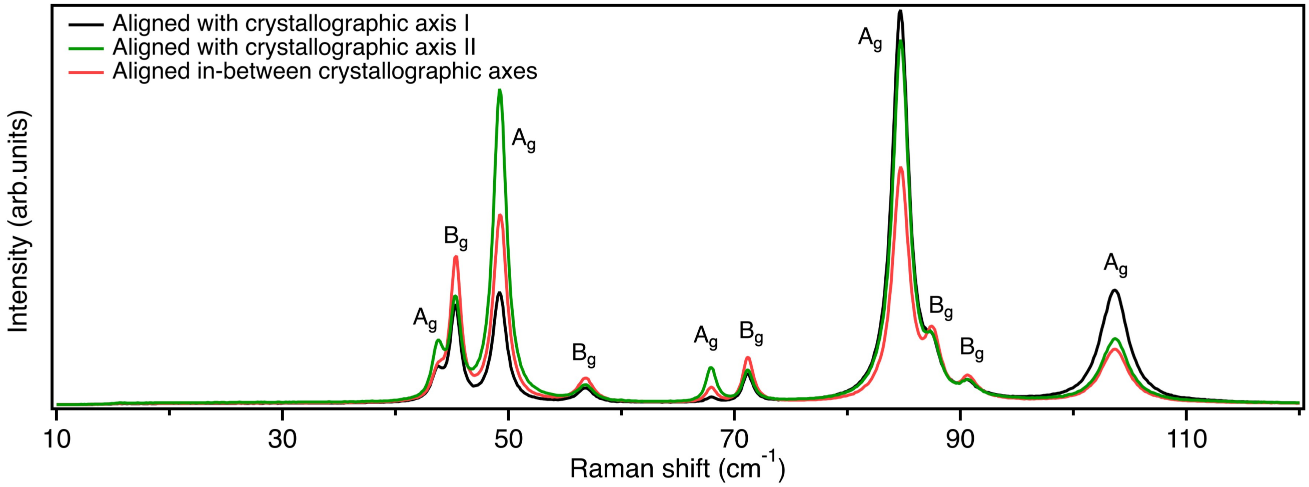

Figure S7: The Raman spectra of ditBu-BTBT at 80 K for three polarization angles. The assigned vibrational symmetry of each peak is noted.

S7 Temperature dependent low-frequency Raman spectroscopy

Figure S8 shows the temperature dependent low-frequency Raman spectroscopy spectra of BTBT, ditBu-BTBT, diC8-BTBT, diPh-BTBT and DNTT from 80 K to 400 K at increments of 10 K.

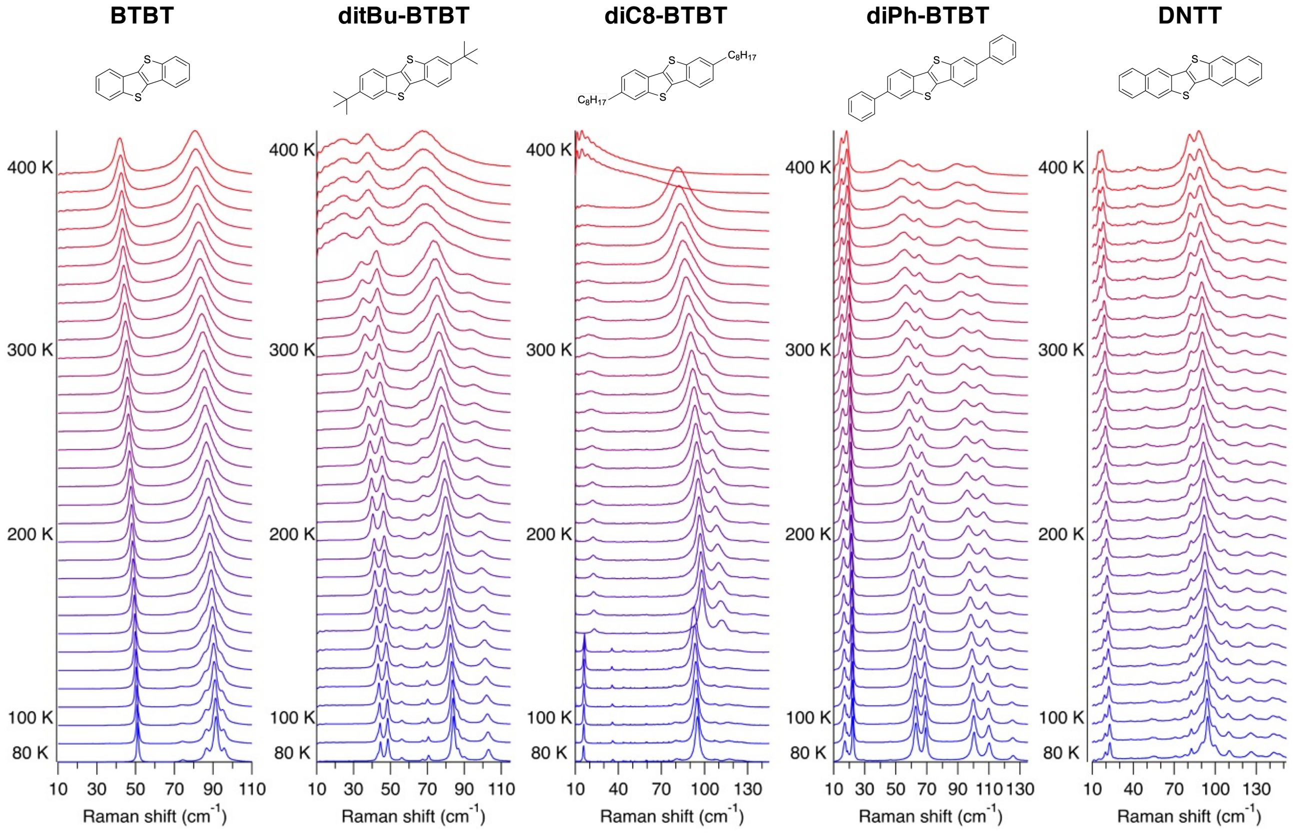

Figure S8: Temperature dependent low-frequency Raman of BTBT, ditBu-BTBT, diC8-BTBT, diPh-BTBT and DNTT. The spectra were normalized and shifted up for clarity. The temperature increment is 10 K.

We extract the temperature dependence of the peaks position and full-width-half-maximum (FWHM) of their lattice vibrations by fitting the spectra according to Section S5

Within each phase, the trends with temperature of the vibrational frequencies and the FWHM is primarily linear due to their dependence on the Bose-Einstein occupation factor, as mentioned in the main text.

We also see an interesting phenomenon in the temperature dependence of the lowest frequency peaks (15-25 cm^{-1}) of diPh-BTBT and DNTT. These peaks are relatively sharp and are weakly dependent on temperature. This means that they are highly populated and have a relatively long lifetime. Since lower frequencies are related to larger amplitude motion, one might

naively correlate it with strong vibrational anharmonicity. We claim this results from a relatively low number of possible phonon decay paths. Concerning the low-frequency region higher frequency lattice vibrations can perform both down-conversion (resulting with lower frequency vibrations) and up-conversion (resulting with higher frequency vibrations) phonon-phonon interactions processes[15, 16]. On the contrary, the lowest frequency vibrations can perform mostly up-conversion as the probability for down conversion decreases with frequency as the density of states below decreases. Other organic crystals show similar behavior where the lower the frequency of the lattice vibration, the sharper and less susceptible to temperature it is [17–19]. Since higher-order terms in the vibrational potential energy surface (i.e., non-parabolic shape) are related to phonon-phonon interaction, our results indicate that the potential energy surfaces of these very-low-frequency lattice modes are more harmonic than that of the higher frequency lattice modes. These very-low-frequency peaks appear in diPh-BTBT and DNTT, and not in BTBT and ditBu-BTBT, probably due to the higher molecular weight and the existence of very low-frequency modes of the isolated molecule. diC8-BTBT also exhibits very-low-frequency peaks, but they do not share the same sharpness and weak temperature dependence. This is probably due to the nature of the phonon-phonon coupling of these modes.

Tables S7-S11 show the values of the slopes for the vibrational frequencies and the FWHM of the lattice vibrations of the five crystals. For the lattice vibrations, which show a quadratic temperature dependence of the FWHM, we calculated the slope for a lower temperature range where the temperature dependence is close to being linear. For diC8-BTBT and DNTT, some slopes' values are absent since the Raman fit confidence is low.

Table S7: Slopes of the temperature dependence of the peaks position and FWHM of BTBT according to linear fit results.

Vibration	Frequency at 80 K (cm^{-1})	Frequency slope ($\frac{cm^{-1}}{100\ K}$)	FWHM slope ($\frac{cm^{-1}}{100\ K}$)
ω_1	51.1	-2.8	1.4
ω_3	74.1	-2.9	3.7
ω_4	86.5	-2.2	4.2
ω_5	91.4	-3.3	2.8
ω_6	95.9	-3.3	4.0

Figure S9 shows the correlation between the values of the slopes of the vibrational frequencies and the FWHM with temperature for each material, compared with their uniaxial thermal

Table S8: Slopes of the temperature dependence of the peaks position and FWHM of ditBu-BTBT (low-temperature phase) according to linear line fit results.

Vibration	Frequency at 80 K (cm^{-1})	Frequency slope ($\frac{cm^{-1}}{100\ K}$)	FWHM slope ($\frac{cm^{-1}}{100\ K}$)
ω_1	43.8	-1.9	2.4
ω_2	45.3	-4.1	2.0
ω_3	49.2	-2.4	1.6
ω_4	56.8	-1.8	3.1
ω_5	67.9	-1.4	1.1
ω_6	71.2	-2.1	2.0
ω_7	84.7	-3.8	4.3
ω_8	87.5	-3.8	2.0
ω_9	90.8	-2.4	4.6
ω_{10}	103.7	-4.0	3.9

Table S9: Slopes of the temperature dependence of the peaks position and FWHM of diC8-BTBT (high-temperature phase) according to linear line fit results.

Vibration	Frequency at 150 K (cm^{-1})	Frequency slope ($\frac{cm^{-1}}{100\ K}$)	FWHM slope ($\frac{cm^{-1}}{100\ K}$)
ω_1	22.4	-0.9	4.5
ω_2	58.2	-8.5	10.2
ω_3	90.8	—	5.1
ω_4	98.3	-7.9	5.6
ω_5	110.8	-10.1	3.3
ω_6	113.5	-5.1	—
ω_7	122.0	—	2.4
ω_8	134.0	-3.0	1.2

Table S10: Slopes of the temperature dependence of the peaks position and FWHM of diPh-BTBT according to linear line fit results.

Vibration	Frequency at 80 K (cm^{-1})	Frequency slope ($\frac{cm^{-1}}{100\ K}$)	FWHM slope ($\frac{cm^{-1}}{100\ K}$)
ω_1	17.1	-0.8	0.4
ω_2	19.0	-0.7	0.7
ω_3	22.4	-1.2	0.4
ω_4	62.8	-2.8	2.8
ω_5	69.2	-1.3	1.2
ω_6	100.3	-3.4	3.2
ω_7	110.0	-3.0	1.9
ω_8	125.5	-2.8	2.2

expansion coefficients. We can see that for diC8-BTBT, one axis with a relatively large range of anisotropy is translated to the values of the slopes where we have similar observations. This correlation shows that the QHA could be good enough to describe the shift in vibrational frequencies with temperature of these materials.

Table S11: Slopes of the temperature dependence of the peaks position and FWHM of DNTT according to linear line fit results.

Vibration	Frequency at 80 K (cm^{-1})	Frequency slope ($\frac{cm^{-1}}{100\ K}$)	FWHM slope ($\frac{cm^{-1}}{100\ K}$)
ω_1	19.5	-1.0	1.4
ω_2	22.9	-1.6	2.6
ω_3	54.3	-2.0	—
ω_4	56.5	-2.5	1.5
ω_5	70.7	-2.6	0.9
ω_6	77.8	-2.1	1.5
ω_7	82.4	-1.8	1.2
ω_8	89.7	-2.4	—
ω_9	94.5	-0.4	1.3
ω_{10}	100.0	-1.3	—
ω_{11}	110.2	-1.8	2.4
ω_{12}	126.2	-0.7	—
ω_{13}	133.9	-2.6	—
ω_{14}	142.7	-1.6	—
ω_{15}	147.2	-1.5	0.3

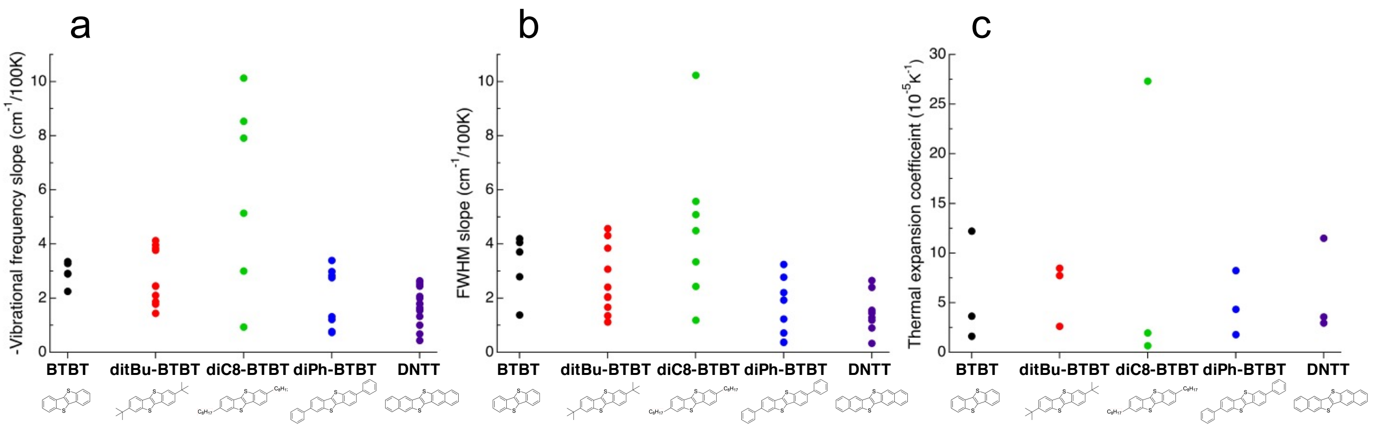

Figure S9: The slopes values of the vibrational frequencies and the FWHM with temperature for each material, compare with their uniaxial thermal expansion coefficients.

S8 DFT calculations

Tables S12 to S16 show the symmetry of calculated vibrational modes at 80 K and assign them to their closest analogue among the experimental modes. In some cases, the assignment is uncertain due to the experimental FWHM of Raman peaks, which tend to convolute modes of similar energy and obscure modes with very small intensities. This effect is more pronounced when more atoms are contained in the unit cell of a given material, and therefore more vibrational degrees of freedom are present. We assign the modes based on peaks proximity and their vibrational symmetry. The experimental vibrational symmetries are extracted according to the procedure described in section S6. Using the intensities for mode assignment is problematic in our case since the calculated intensities consider the 3D Raman cross-section while, as mentioned in the main text, in our measurements, the polarization is rotating within a 2D plane parallel to the crystal surface. For diPh-BTBT, according to the Raman tensors form associated with its crystal symmetry and the measured crystal plane, only the A_g and the B_{3g} modes are detectable (the B_{1g} and B_{2g} modes are not, see section S14 for more details).

The agreement between the measured and calculated Raman peaks is relatively good. The average and standard deviations of the differences between the measured and calculated Raman peaks position of BTBT, ditBu-BTBT, diC8-BTBT, diPh-BTBT, and DNTT are 3.3±2.3, 3.8±3.3, 5.0±4.2, 4.5±3.0, and 2.3±2.1 cm^{-1} respectively. These values are common for this type of calculations [17, 20–22]. The modes eigenvectors for each material are attached to this publication as media (.xsf) files.

Figure S10, shows the ability of Quasi Harmonic Approximation calculations (QHA) to qualitatively reproduce the experimentally observed redshift of vibrational peaks at higher temperatures. Thus, by using the QHA, we can account for some of the observed anharmonic effects.

Table S12: Mode assignment: The calculated (and experimental) frequency of each of the first six Raman modes of BTBT, alongside the calculated (and experimental) symmetry of each mode and its relative intensity. The calculation was performed on a SCXRD structure of BTBT collected at low T (100K) after a full computational relaxation of atomic positions.

	ω_1	ω_2	ω_3	ω_4	ω_5	ω_6
Freq. (exp) (cm^{-1})	44 (52)	51 (53)	72 (75)	82 (88)	95 (93)	96 (97)
Symmetry (exp)	A_g (A_g)	B_g	A_g (A_g)	B_g (B_g)	A_g (A_g)	B_g (B_g)
Rel. Intensity	0.188	0.053	0.260	0.130	1.000	0.295

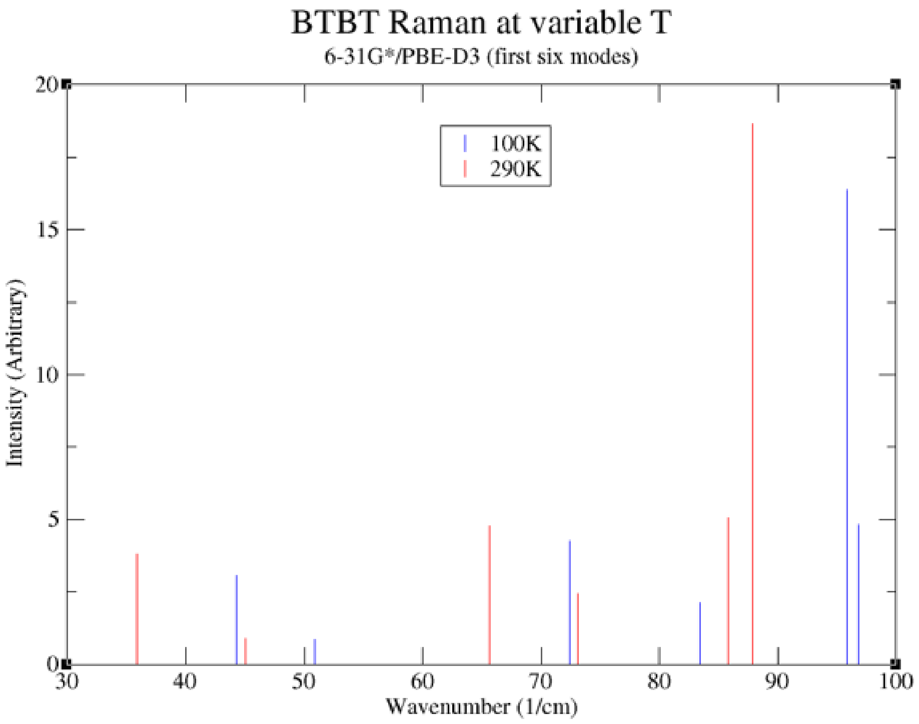

Figure S10: Quasi-Harmonic Approximation calculated Raman peaks from computationally relaxed structures constrained by experimental lattice vectors collected at 290K (red) and 100K (blue). This shows the ability of the Quasi-Harmonic Approximation to capture some anharmonic effects, such as the T-dependent redshift in the position of the peaks.

Table S13: Mode assignment: The calculated (and experimental) frequency of each of the first ten Raman modes of ditBu-BTBT, alongside the calculated (and experimental) symmetry of each mode and its relative intensity. The calculation was performed on a SCXRD structure of ditBu-BTBT after a full computational relaxation of atomic positions.

	ω_1	ω_2	ω_3	ω_4	ω_5
Freq. (exp) (cm^{-1})	40 (44)	41 (47)	47 (50)	52 (57)	65 (68)
Symmetry (exp)	A_g (A_g)	B_g (B_g)	A_g (A_g)	B_g (B_g)	A_g (A_g)
Rel. Intensity	0.303	0.065	0.181	0.045	0.106
	ω_6	ω_7	ω_8	ω_9	ω_{10}
Freq. (exp) (cm^{-1})	75 (72)	86 (86)	89 (89)	94 (92)	107 (95)
Symmetry (exp)	B_g (B_g)	A_g (A_g)	B_g (B_g)	B_g (B_g)	A_g (A_g)
Rel. Intensity	0.055	1.000	0.028	0.175	0.930

Table S14: Mode assignment: The calculated (and experimental) frequency of the Raman modes of diC8-BTBT below 130 cm^{-1}, alongside the calculated (and experimental) symmetry of each mode and its relative intensity. The calculation was performed on a SCXRD structure of diC8-BTBT after a full computational relaxation of atomic positions.

	ω_1	ω_2	ω_3	ω_4	ω_5	ω_6
Freq. (exp) (cm^{-1})	16 (27)	19 (23)	41	45	50	57 (58)
Symmetry (exp)	A_g (A_g)	B_g (B_g)	B_g	A_g	B_g	A_g (A_g)
Rel. Intensity	0.014	0.013	0.073	0.030	0.012	0.042
	ω_7	ω_8	ω_9	ω_{10}	ω_{11}	ω_{12}
Freq. (exp) (cm^{-1})	73	74	87 (90)	95	97	98 (98)
Symmetry (exp)	A_g	B_g	A_g (A_g)	B_g	B_g	A_g (A_g)
Rel. Intensity	0.030	0.016	0.232	0.028	0.084	0.131
	ω_{13}	ω_{14}	ω_{15}	ω_{16}	ω_{17}	ω_{18}
Freq. (exp) (cm^{-1})	104 (110)	108 (113)	113	115	121 (122)	122 (134)
Symmetry (exp)	A_g (A_g)	B_g (B_g)	B_g	A_g	A_g (A_g)	B_g (B_g)
Rel. Intensity	0.255	0.179	0.092	1.000	0.025	0.072

Table S15: Mode assignment: The calculated (and experimental) frequency of the Raman modes of diPh-BTBT below 130 cm^{-1}, alongside the calculated (and experimental) symmetry of each mode and its relative intensity. The calculation was performed on a SCXRD structure of diPh-BTBT after a full computational relaxation of atomic positions.

	ω_1	ω_2	ω_3	ω_4	ω_5	ω_6	ω_7
Freq. (exp) (cm^{-1})	19 (17)	29 (22)	31	44	70 (62)	71	72 (69)
Symmetry (exp)	B_{3g} (B_{3g})	A_g (A_g)	B_{2g}	B_{1g}	A_g (A_g)	B_{2g}	A_g (A_g)
Rel. Intensity	0.011	0.165	0.041	0.030	0.032	0.122	1.000
	ω_8	ω_9	ω_{10}	ω_{11}	ω_{12}	ω_{13}	ω_{14}
Freq. (exp) (cm^{-1})	75	76	81	84	95	100	108
Symmetry (exp)	B_{2g}	B_{3g}	B_{1g}	A_g	B_{2g}	B_{3g}	B_{2g}
Rel. Intensity	0.041	0.005	0.016	0.081	0.027	0.038	0.003
	ω_{15}	ω_{16}	ω_{17}	ω_{18}	ω_{19}	ω_{20}	ω_{21}
Freq. (exp) (cm^{-1})	109 (100)	113 (110)	115	117	124.0	124.2 (125)	132
Symmetry (exp)	A_g (A_g)	B_{3g} (B_{3g})	B_{1g}	A_g	B_{1g}	B_{3g} (B_{3g})	B_{2g}
Rel. Intensity	0.519	0.119	0.224	0.397	0.027	0.132	0.022

Table S16: Mode assignment: The calculated (and experimental) frequency of the Raman modes of DNTT below 150 cm^{-1}, alongside the calculated (and experimental) symmetry of each mode and its relative intensity. The calculation was performed on a SCXRD structure of DNTT after a full computational relaxation of atomic positions.

	ω_1	ω_2	ω_3	ω_4	ω_5
Freq. (exp) (cm^{-1})	12 (20)	23 (23)	34	55.2 (54)	55.5 (56)
Symmetry (exp)	A (A)	B (B)	A	A (A)	A (A)
Rel. Intensity	0.009	0.120	0.019	0.130	0.029
	ω_6	ω_7	ω_8	ω_9	ω_{10}
Freq. (exp) (cm^{-1})	69 (71)	75 (78)	82 (82)	83	90 (90)
Symmetry (exp)	B (B)	B (B)	A (A)	B	A (A)
Rel. Intensity	0.007	0.046	0.075	0.009	0.110
	ω_{11}	ω_{12}	ω_{13}	ω_{14}	ω_{15}
Freq. (exp) (cm^{-1})	94 (94)	98 (100)	106 (110)	121	126
Symmetry (exp)	A (A)	B (B)	B (B)	B	A
Rel. Intensity	1.000	0.260	0.100	0.011	0.070
	ω_{16}	ω_{17}	ω_{18}	ω_{19}	ω_{20}
Freq. (exp) (cm^{-1})	128 (126)	130 (134)	139 (143)	144 (147)	151
Symmetry (exp)	B (B)	A (A)	A (A)	B (B)	A
Rel. Intensity	0.220	0.210	0.400	0.037	0.027

S9 Temperature dependent polarization-orientation (PO) Raman measurements

Figures S11-S15 present the raw data we obtain from the PO Raman measurements for both parallel and perpendicular configurations for single crystals of BTBT, ditBu-BTBT, diC8-BTBT, diPh-BTBT, and DNTT.

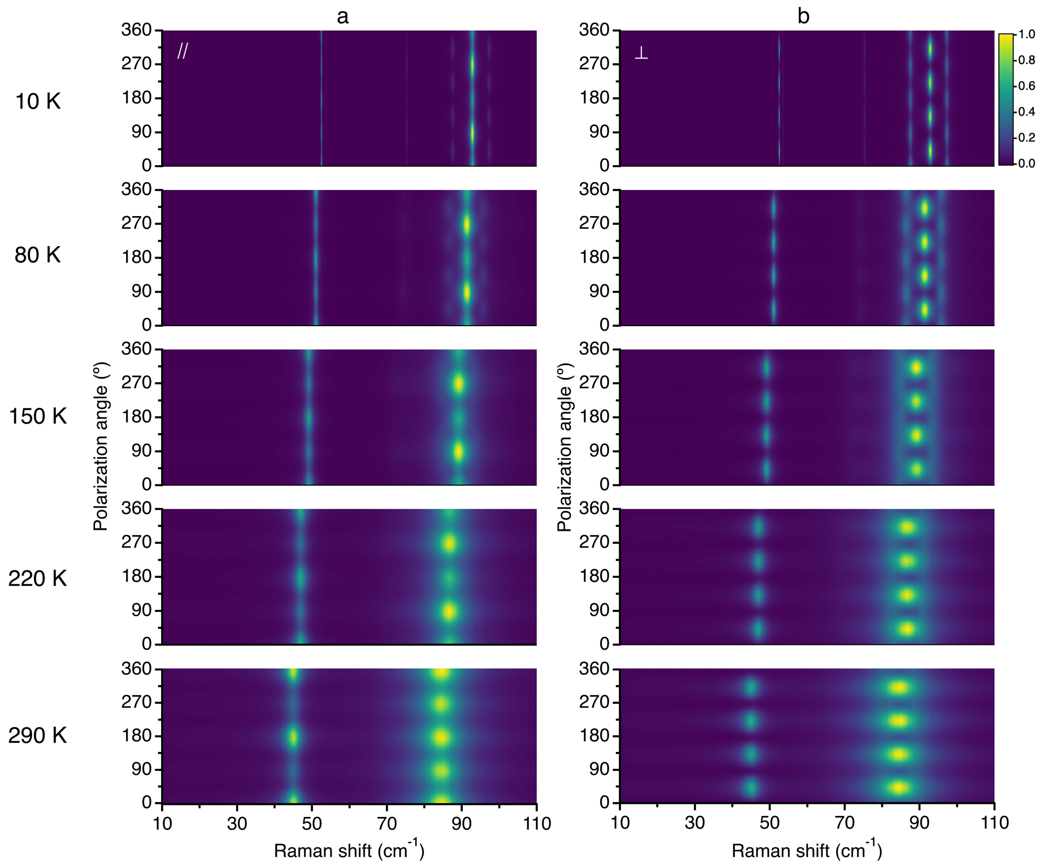

Figure S11: Raw PO Raman of BTBT in (a) parallel and (b) perpendicular configurations at 10 K, 80 K, 150 K, 220 K and 290 K.

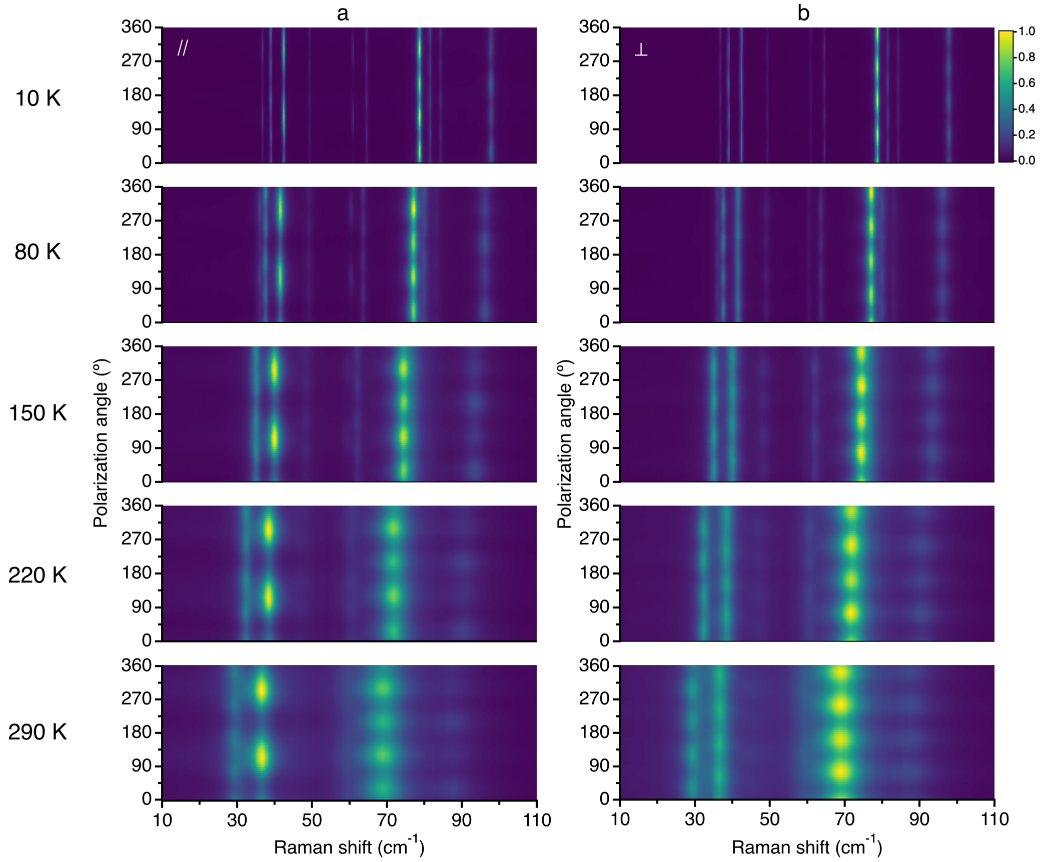

Figure S12: Raw PO Raman of ditBu-BTBT in (a) parallel and (b) perpendicular configurations at 10 K, 80 K, 150 K, 220 K and 290 K.

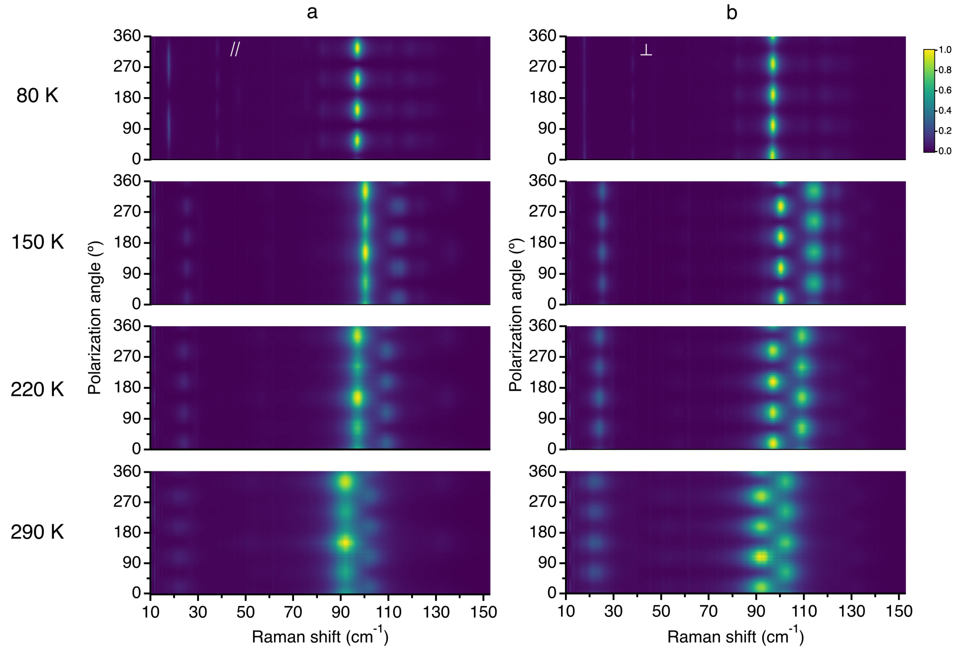

Figure S13: Raw PO Raman of diC8-BTBT in (a) parallel and (b) perpendicular configurations at 80 K, 150 K, 220 K and 290 K.

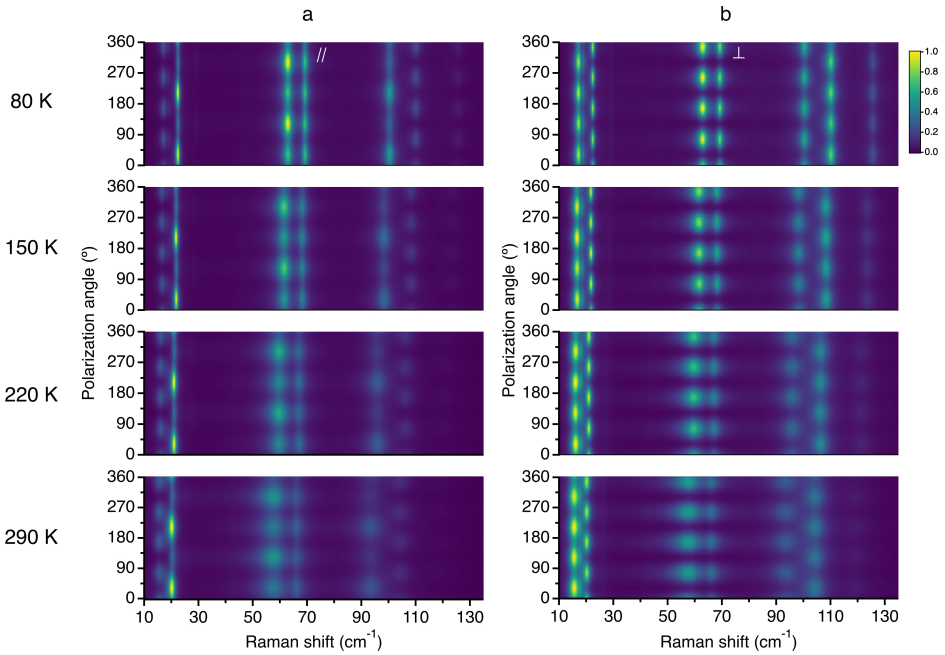

Figure S14: Raw PO Raman of diPh-BTBT in (a) parallel and (b) perpendicular configurations at 80 K, 150 K, 220 K and 290 K.

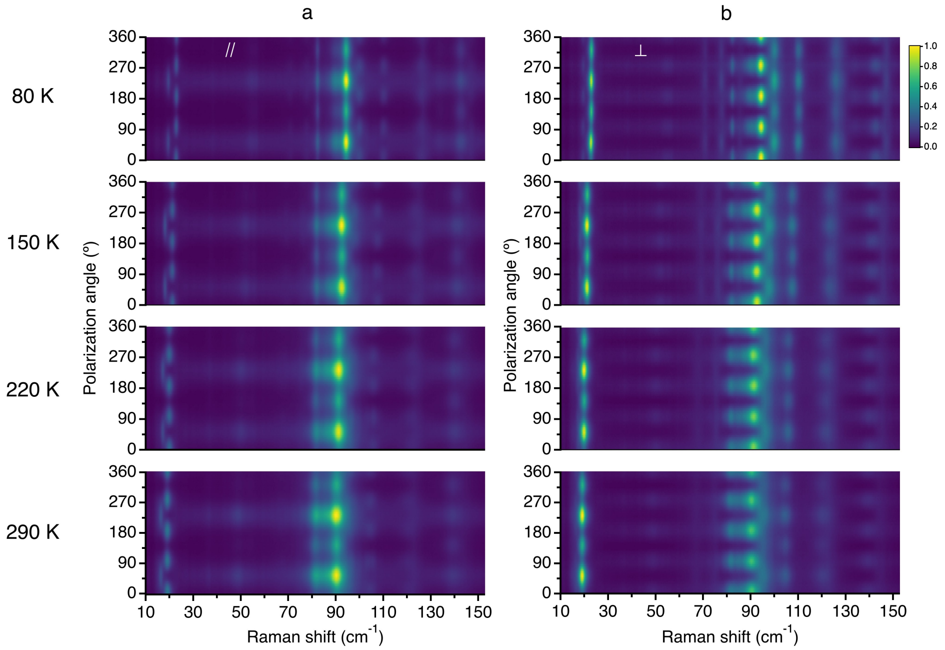

Figure S15: Raw PO Raman of DNTT in (a) parallel and (b) perpendicular configurations at 80 K, 150 K, 220 K and 290 K.

S10 Temperature dependent polarization-orientation (PO) Raman of silicon

The PO response of a given mode is dictated by is Raman tensor. The form/shape of this Raman tensor is dictated by its vibrational symmetry and crystal structure. In the harmonic picture, the vibrational symmetries and the Raman tensors are not expected to change with temperature. Therefore, a temperature-dependent PO response is an indication of an anharmonic effect in the lattice dynamics of the crystals. This anharmonic effect is shown in the main text for BTBT and in our previous publication[17]. Figure S16 presents the PO Raman measurements of a (100) silicon wafer at 10 K and 300 K with the analysis of the polarization dependence of the integrated intensity of the prominent Raman peak (the TO phonon at around 520 cm^{-1}). We performed the measurements and analysis similar to the organic crystals presented in the main text using the same optical setup. We present these results as an example for an inorganic material that shows that the PO response is temperature independent, thus following the harmonic picture. Furthermore, according to theory, the intensity of the prominent Raman peak should go down to zero at the minimum point [23]. In our measurement, the peak intensity drops to about 1% of its maximum intensity, showing the minimal leakage of our system.

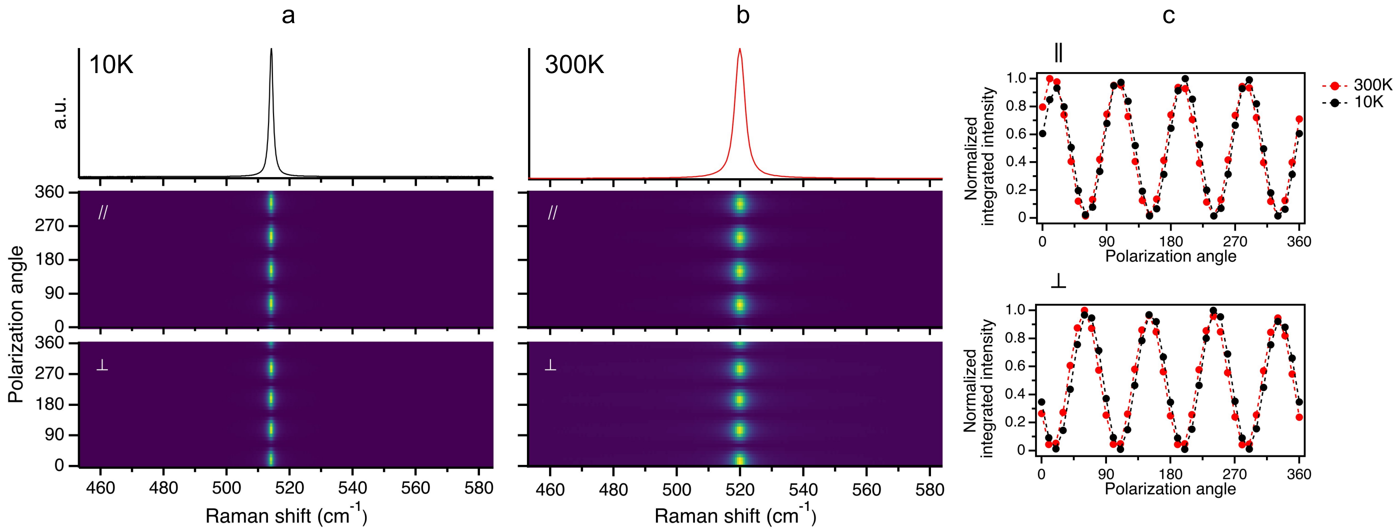

Figure S16: Temperature dependence of the PO Raman of silicon. (a) and (b) presents the PO Raman measurement of silicon at 10 K and 300 K, respectively. The middle and bottom panels present the measurements in parallel and perpendicular configurations, respectively. The top panel presents the unpolarized spectrum (sum over all angles). (c) presents the polarization dependence of the integrated intensity of the prominent Raman peak. The top and bottom panels present the results for parallel and perpendicular configurations, respectively.

S11 Fitting the PO response of BTBT at 10 K

Figure S18a presents the contour plots of the PO Raman measurement of BTBT at 10 K in parallel and perpendicular configurations along with the unpolarized Raman spectrum. The measurement was performed similarly to the method described in the main text. To extract the polarization dependence of the integrated intensity of each peak, we choose the polarization angle where each peak is most pronounced (for example, see Figure S17) and extract its position and width by fitting the spectra to a multi-damped Lorentz oscillator (see section S5). Afterward, we fit the Raman spectra at each polarization angle by fixing the position and width of each mode we already extracted and allowing only its intensity as a varying parameter. the spectra at each polarization angle to a multi-damped Lorentz oscillator (see section S5). The dotted lines in Figure S18b show the integrated intensity of each damped Lorentz oscillator with respect to the excitation polarization angle. Finally, we perform a global fit to the integrated intensity in both parallel and perpendicular configurations to a model [24] based on the harmonic approximation [25],

$$I \propto |e_i \tilde{R} e_s|^2 \tag{2}$$

Where I is the polarization-dependent integrated intensity, e_i and e_s are the incident and scattered polarization vectors respectively, and $\tilde{R}$ is the effective Raman tensor. This tensor takes into account several corrections to accurately extract the Raman tensors, such as the effects of birefringence and the vibrational frequencies on the scattering cross-section (for more details regarding the fitting process, see Ref.[17]).

To extract the form of the Raman tensors, as well as the expected number of lattice modes and their vibrational symmetry, we use factor group analysis [26] to the relevant space group (monoclinic, $P2_1/c$). For BTBT, factor group analysis predicts 6 Raman-active lattice libration modes, 3 A_g and 3 B_g modes with the Raman tensor form:

$$R_{A_g} = \begin{pmatrix} a & 0 & e \\ 0 & b & 0 \\ e & 0 & c \end{pmatrix}, R_{B_g} = \begin{pmatrix} 0 & d & 0 \\ d & 0 & f \\ 0 & f & 0 \end{pmatrix} \tag{3}$$

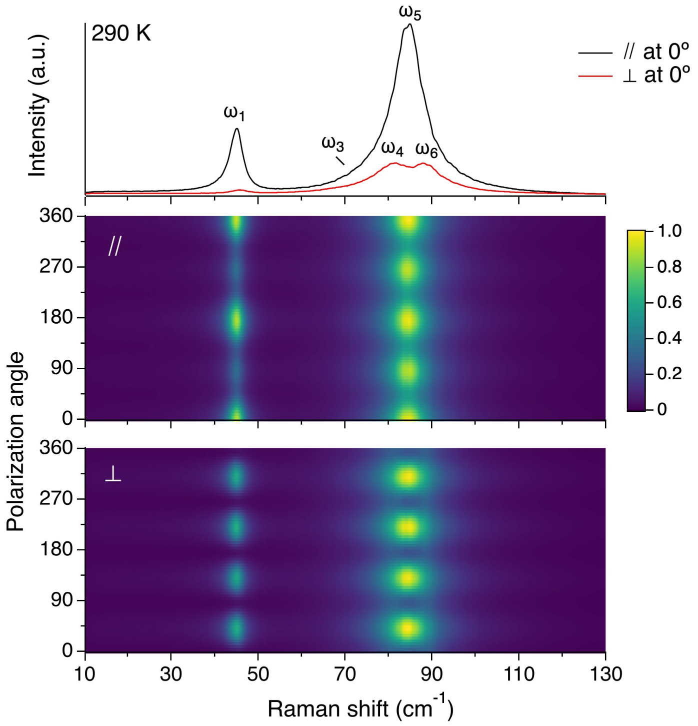

Figure S17: PO Raman measurement of BTBT at 290 K. The top panel shows the Raman spectra in parallel and perpendicular configuration at 0° where different peaks are pronounced. The middle and bottom panels show the polarization-dependent Raman in parallel and perpendicular configuration respectively for all angles.

From our Raman measurement, we obtained the exact number of six Raman active modes (see top panel of Figure S18a). Figure S18b shows the obtained results from this fitting procedure of the lattice vibrations of BTBT at 10 K. The fitting results show that ω_1, ω_3, and ω_5 are A_g modes while ω_4 and ω_6 are B_g modes. The intensity of ω_2 was too weak to extract its polarization dependence reliably.

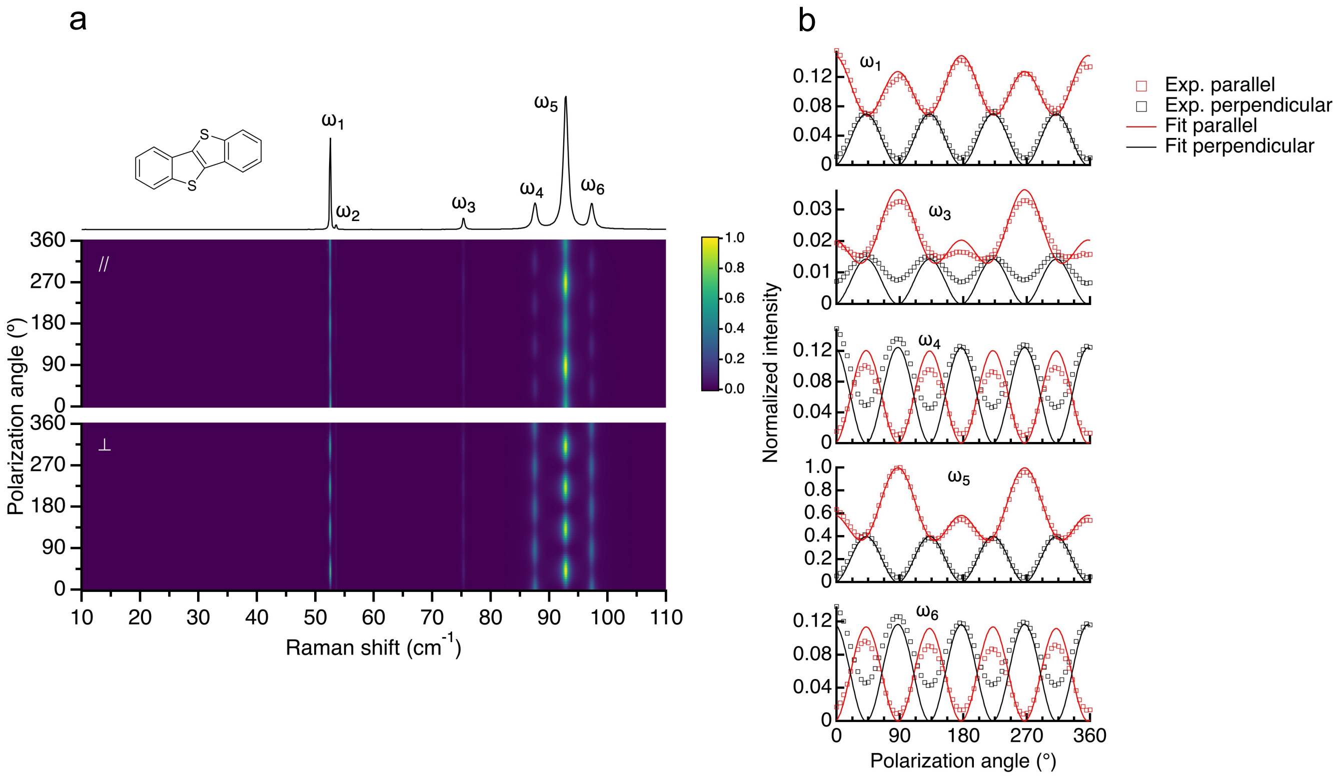

Figure S18: BTBT PO Raman at 10 K. (a) Unpolarized Raman spectra (top panel) and the PO dependence in parallel (mid panel) and perpendicular configuration (lower panel). (b) The fit results of the polarization dependence integrated intensity of each peak.

S12 Single-crystal XRD of the low-temperature phase of diC8-BTBT

We perform single-crystal XRD for diC8-BTBT at 100 K. A small piece of diC8-BTBT was glossed with Paratone oil, mounted on a lithographic Mitegen loop, and flash cooled to 100K. Several attempts were made using different crystals. Most crystals shutter to pieces when introduced to 100K (Figure S19). Data were collected from a tiny piece that survived the temperature change and still diffracted. Data were collected on a Rigaku Synergy-R diffractometer equipped with HyPix-Arc 150 detector, CuKα (λ=1.54184 Å). Structure solved with SHELXT[27] program and refined with SHELXL[13] and OLEX2[28]. All non-hydrogen atoms were further refined with anisotropic displacement coefficients. Hydrogen atoms were assigned isotropic displacement coefficients, and their coordinates were allowed to ride on their respective carbons.

The results of this measurement are shown in Table S6. More crystal data: $C_{30}H_{40}S_2$ (Figure S20), colorless plate 0.208x0.083x0.025 mm^3, Triclinic P-1, from 15247 reflections from 25°, T=100(2)K, Z=2, Fw=464.74, Dc=1.241 Mg.m^{-3}, μ=2.036 mm^{-1}. $-8 \leq h \leq 8$, $-5 \leq k \leq 5$, $-27 \leq l \leq 27$; R-int =0.0517, completeness 98.8%. Full matrix least-squares refinement based on F^2 on 261 parameters with no restraints gave final R_1= 0.0982 (based on F^2) wR_2= 0.3116 for data with I>2α(I), and R_1= 0.1372 (based on F^2) wR_2= 0.3504 for all data, on 2579 reflections. Goodness-of-fit on F^2 = 1.244. Largest electron density peak 1.166 eÅ^{-3} and largest hole –0.622 eÅ^{-3}.

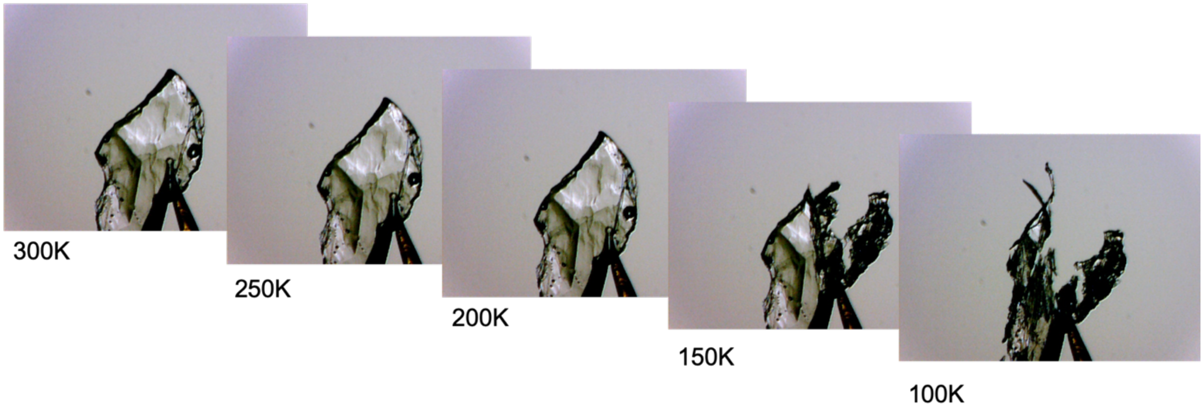

Figure S19: Pictures of the measured diC8-BTBT crystal before each temperature measurement. At 100K the crystals shuttered to tiny pieces and did not diffract anymore in most cases.

Figure S21 shows diC8-BTBT crystal packing comparison between the new triclinic structure, measured at 100K and presented in this work, and the known published monoclinic structure,

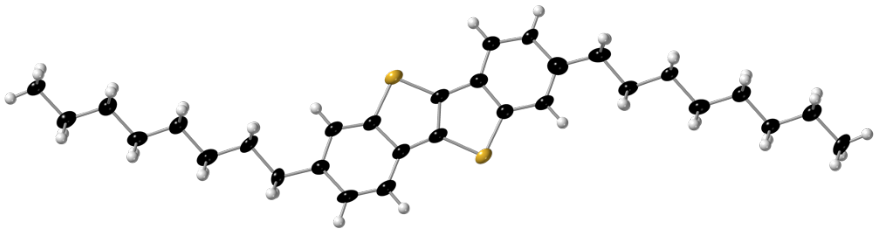

Figure S20: ORTEP presentation of diC8-BTBT at 100K. Thermal ellipsoids 50% probability. Carbon-black, Sulphur-yellow, hydrogen-white spheres.

CCDC-679293, measured at 293K. Figure S21a and b presents the *bc* and *ab* planes respectively of the monoclinic structure. Figure S21c and d presents the *bc* and *ab* planes respectively of the triclinic structure. In the monoclinic phase, the molecules are stuck on top of each other, but the aromatic molecule part has alternating rotation, which does not exist in the triclinic structure. The carbohydride side chains seem more stretched out in the triclinic case, and the thermal ellipsoids are smaller because of the measurement temperature.

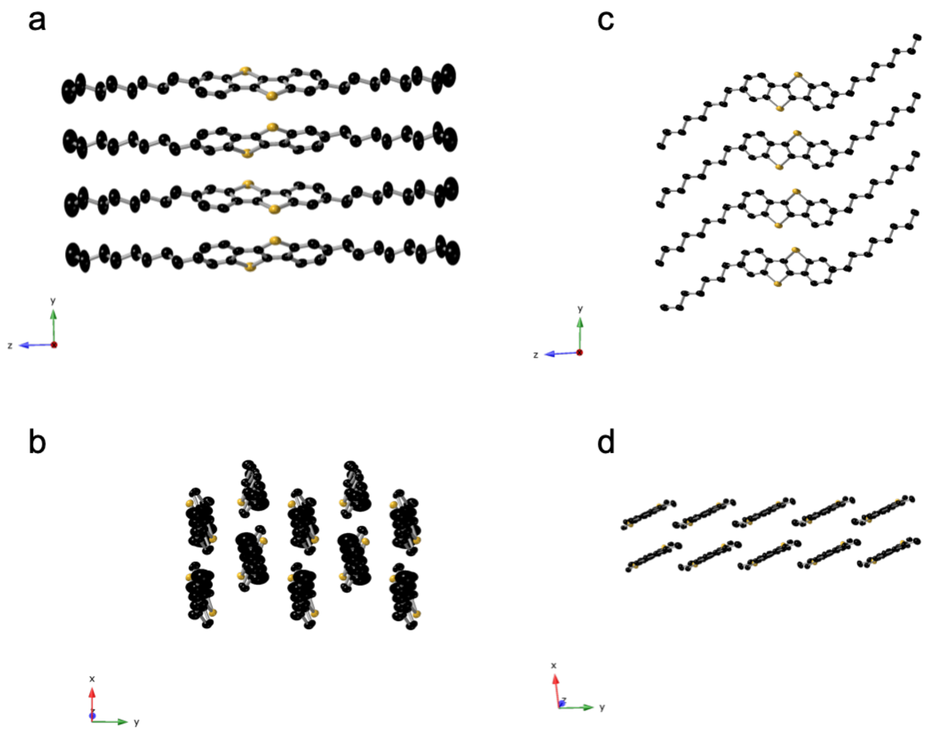

Figure S21: Crystal packing comparison of diC8-BTBT at 293K (monoclinic) and 100K (triclinic). Thermal ellipsoids 50% probability. Carbon-black, Sulphur-yellow, hydrogens are omitted for clarity. The monoclinic structure (a) *bc* plane and (b) *ab* plane; The triclinic structure (c) *bc* plane and (d) *ab* plane.

S13 Polarization-orientation (PO) Raman of chloroform

Figure S22 presents the PO Raman measurement of liquid chloroform at room temperature. The measurement was performed similarly to the organic crystals presented in the main text using the same optical setup. Since there is no long-range order in a liquid, we expect the measurement to be polarization independent. This is why measuring the PO response of a liquid is helpful as a test for the system response to the change in polarization angle. Our results show that the PO response of the vibrations of chloroform is completely polarization-independent - proving our systems response is minimal.

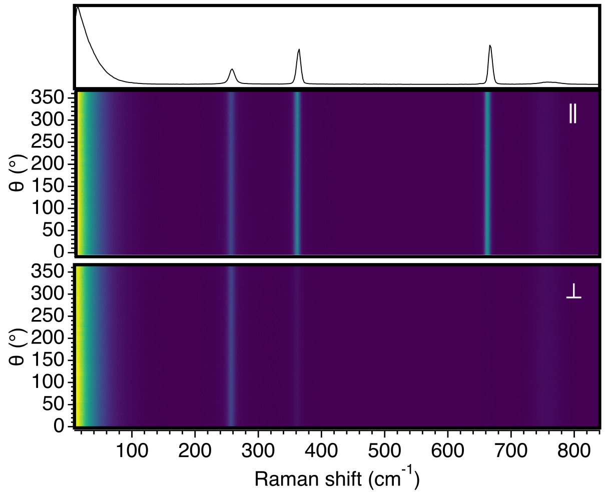

Figure S22: Polarization dependent measurement of liquid chloroform in parallel and perpendicular configurations. The top panel shows a typical spectrum in the parallel configuration.

S14 Factor group analysis

Factor group analysis is a common practice where the factor group of a crystal space group is used to assign to each peak in the spectrum a single irreducible representation (irrep, e.g., A_g, T_{2g}, A_u...) out of those made available by the reduced mechanical representations. We use factor group analysis to extract the number of Raman active modes, their vibrational symmetries, and Raman tensor form. We do that while assuming the rigid-molecule approximation, meaning the molecules are considered to be rigid bodies (i.e., no internal degrees of freedom). In some cases, the number of observed low-frequency Raman peaks is larger than that predicted by factor group analysis. This usually occurs once a molecule becomes long and flexible enough, so the single isolated molecule has low-frequency intramolecular vibrations [29, 30]. These vibrations in the crystal form mix with the intermolecular modes, creating more peaks in the low-frequency range. As a result, the eigenvectors of the low-frequency modes will have a combination of inter and intramolecular motion [31, 32] (not to be confused with mode coupling).

ditBu-BTBT and diC8-BTBT have the same crystal structure ($P2P1/c$) and Wyckoff position as BTBT (as presented in Section S11). Thus, factor group analysis produces the same results: 6 Raman active librational modes, 3 A_g and 3 B_g modes. For both crystals, we observe more Raman active modes than predicted.

The space group of diPh-BTBT is *Pbca* (No. 61), where the atoms occupy the c8 positions. Accordingly, factor group analysis predicts 24 Raman active modes. 3 A_g, 3 B_{1g}, 3 B_{2g} and 3 B_{3g} translational modes, and the same set of librational modes. The Raman tensor form of these modes are:

$$R_{A_g} = \begin{pmatrix} a & 0 & 0 \\ 0 & b & 0 \\ 0 & 0 & c \end{pmatrix}, R_{B_{1g}} = \begin{pmatrix} 0 & d & 0 \\ d & 0 & 0 \\ 0 & 0 & 0 \end{pmatrix}, R_{B_{2g}} = \begin{pmatrix} 0 & 0 & e \\ 0 & 0 & 0 \\ e & 0 & 0 \end{pmatrix}, R_{B_{3g}} = \begin{pmatrix} 0 & 0 & 0 \\ 0 & 0 & f \\ 0 & f & 0 \end{pmatrix} \quad (4)$$

Since our incident light is perpendicular to the [100] direction, the polarization of the light is rotating in the (100) plane. Thus, we can measure only the y and z components of the Raman tensors. That reduces the predicted number of Raman active modes to 12 as we should not see the B_{1g} and B_{2g} modes. In our measurement, we observe 8 peaks. The reasons for the lower

number of peaks observed than predicted, can be from low scattering cross-section of some modes or from unresolved peaks since the lowest measured temperature was 80 K.

The space group of DNTT is $P2_1$ (No. 4), where the atoms occupy the a2 positions. Accordingly, factor group analysis predicts 12 Raman active modes. 3 A and 3 B translational modes, and the same set of librational modes. The Raman tensor form of these modes are:

$$R_{A_g} = \begin{pmatrix} a & 0 & e \\ 0 & b & 0 \\ e & 0 & c \end{pmatrix}, R_{B_{1g}} = \begin{pmatrix} 0 & d & 0 \\ d & 0 & f \\ 0 & f & 0 \end{pmatrix} \tag{5}$$

Similar to diPh-BTBT, we can measure only the y and z components of the Raman tensors, but in this case we are not restricted by the Raman tensors form, since we observe 15 Raman active modes at 80 K.

References

(1) Vyas, V. S.; Gutzler, R.; Nuss, J.; Kern, K.; Lotsch, B. V. *CrystEngComm* **2014**, *16*, 7389–7392.

(2) Niebel, C. et al. *Journal of Materials Chemistry C* **2015**, *3*, 674–685.

(3) Matsumura, M.; Muranaka, A.; Kurihara, R.; Kanai, M.; Yoshida, K.; Kakusawa, N.; Hashizume, D.; Uchiyama, M.; Yasuike, S. *Tetrahedron* **2016**, *72*, 8085–8090.

(4) Schweicher, G. et al. *Advanced Materials* **2015**, *27*, 3066–3072.

(5) Chung, H.; Dudenko, D.; Zhang, F.; D'Avino, G.; Ruzié, C.; Richard, A.; Schweicher, G.; Cornil, J.; Beljonne, D.; Geerts, Y.; Diao, Y. *Nature Communications* **2018**, *9*, 1–12.

(6) Izawa, T.; Miyazaki, E.; Takimiya, K. *Advanced Materials* **2008**, *20*, 3388–3392.

(7) Yamamoto, T.; Takimiya, K. *Journal of the American Chemical Society* **2007**, *129*, 2224–2225.

(8) Shinamura, S.; Osaka, I.; Miyazaki, E.; Nakao, A.; Yamagishi, M.; Takeya, J.; Takimiya, K. *Journal of the American Chemical Society* **2011**, *133*, 5024–5035.

(9) Macrae, C. F.; Bruno, I. J.; Chisholm, J. A.; Edgington, P. R.; McCabe, P.; Pidcock, E.; Rodriguez-Monge, L.; Taylor, R.; Van De Streek, J.; Wood, P. A. *Journal of Applied Crystallography* **2008**, *41*, 466–470.

(10) Macrae, C. F.; Edgington, P. R.; McCabe, P.; Pidcock, E.; Shields, G. P.; Taylor, R.; Towler, M.; Van De Streek, J. *Journal of Applied Crystallography* **2006**, *39*, 453–457.

(11) *Rigaku Oxford Diffraction* **2019**.

(12) Farrugia, L. J. *Journal of Applied Crystallography* **2012**, *45*, 849–854.

(13) Sheldrick, G. M. *Acta Crystallographica Section C: Structural Chemistry* **2015**, *71*, 3–8.

(14) Banks, P. A.; Maul, J.; Mancini, M. T.; Whalley, A. C.; Erba, A.; Ruggiero, M. T. *Journal of Materials Chemistry C* **2020**, *8*, 10917–10925.

(15) Lan, T.; Li, C. W.; Niedziela, J. L.; Smith, H.; Abernathy, D. L.; Rossman, G. R.; Fultz, B. *Physical Review B - Condensed Matter and Materials Physics* **2014**, *89*, 54306.

(16) Lan, T.; Li, C. W.; Fultz, B. *Physical Review B - Condensed Matter and Materials Physics* **2012**, *86*, 134302.

(17) Asher, M.; Angerer, D.; Korobko, R.; Diskin-Posner, Y.; Egger, D. A.; Yaffe, O. *Advanced Materials* **2020**, *32*, 1908028.

(18) Rivalta, A.; Salzillo, T.; Venuti, E.; Della Valle, R. G.; Sokolovič, B.; Werzer, O.; Brillante, A. *ACS Omega* **2018**, *3*, 9564–9571.

(19) Socci, J.; Salzillo, T.; Della Valle, R. G.; Venuti, E.; Brillante, A. *Solid State Sciences* **2017**, *71*, 146–151.

(20) Zaczek, A. J.; Catalano, L.; Naumov, P.; Korter, T. M. *Chemical Science* **2019**, *10*, 1332–1341.

(21) Bedoya-Martínez, N.; Schrode, B.; Jones, A. O. F.; Salzillo, T.; Ruzié, C.; Demitri, N.; Geerts, Y. H.; Venuti, E.; Della Valle, R. G.; Zojer, E.; Resel, R. *The Journal of Physical Chemistry Letters* **2017**, *8*, 3690–3695.

(22) Vener, M. V.; Parashchuk, O. D.; Kharlanov, O. G.; Maslennikov, D. R.; Dominskiy, D. I.; Yu. Chernyshov, I.; Yu. Paraschuk, D.; Yu. Sosorev, A. *Advanced Electronic Materials* **2021**, *7*, 2001281.

(23) Lu, Z. Q.; Quinn, T.; Reehal, H. S. *Journal of Applied Physics* **2005**, *97*, 33512.

(24) Kranert, C.; Sturm, C.; Schmidt-Grund, R.; Grundmann, M. *Physical Review Letters* **2016**, *116*, 127401.

(25) Cardona, M.; Yu, P., *Fundamentals of Semiconductors*; Springer: 2010, pp 375–380.

(26) Laboratories, B. T.; Hill, M.; Bauman, R. P. *Journal of Raman Spectroscopy* **1981**, *10*, 253–290.

(27) Sheldrick, G. M. *Acta Crystallographica Section A: Foundations of Crystallography* **2015**, *71*, 3–8.

(28) Dolomanov, O. V.; Bourhis, L. J.; Gildea, R. J.; Howard, J. A.; Puschmann, H. *Journal of Applied Crystallography* **2009**, *42*, 339–341.

(29) Abdulla, M.; Refson, K.; Friend, R. H.; Haynes, P. D. *Journal of Physics Condensed Matter* **2015**, *27*, 375402.

(30) Da Silva Filho, D. A.; Kim, E. G.; Brédas, J. L. *Advanced Materials* **2005**, *17*, 1072–1076.

(31) Zhang, F.; Wang, H. W.; Tominaga, K.; Hayashi, M. *Wiley Interdisciplinary Reviews: Computational Molecular Science* **2016**, *6*, 386–409.

(32) Burgos, E.; Bonadeo, H.; D'Alessio, E. *The Journal of Chemical Physics* **1976**, *65*, 2460–2466.
